# CLK2 Condensates Reorganize Nuclear Speckles and Induce Intron Retention

**DOI:** 10.1002/advs.202309588

**Published:** 2024-08-09

**Authors:** Bing Wang, Jing Li, Yanyang Song, Xuhui Qin, Xia Lu, Wei Huang, Chentai Peng, Jinxia Wei, Donghui Huang, Wei Wang

**Affiliations:** ^1^ Department of Human Anatomy School of Basic Medicine Tongji Medical College Huazhong University of Science and Technology Wuhan 430070 China; ^2^ Department of Integrated Traditional Chinese and Western Medicine Tongji Hospital Tongji Medical College Huazhong University of Science and Technology Wuhan 430030 China; ^3^ Institute of Reproduction Health Research Tongji Medical College Huazhong University of Science and Technology Wuhan 430030 China

**Keywords:** CLK2, condensates, heat shock, intron retention, nuclear speckles

## Abstract

Intron retention (IR) constitutes a less explored form of alternative splicing, wherein introns are retained within mature mRNA transcripts. This investigation demonstrates that the cell division cycle (CDC)‐like kinase 2 (CLK2) undergoes liquid–liquid phase separation (LLPS) within nuclear speckles in response to heat shock (HS). The formation of CLK2 condensates depends on the intrinsically disordered region (IDR) located within the N‐terminal amino acids 1‐148. Phosphorylation at residue T343 sustains CLK2 kinase activity and promotes overall autophosphorylation, which inhibits the LLPS activity of the IDR. These CLK2 condensates initiate the reorganization of nuclear speckles, transforming them into larger, rounded structures. Moreover, these condensates facilitate the recruitment of splicing factors into these compartments, restricting their access to mRNA for intron splicing and promoting the IR. The retained introns lead to the sequestration of transcripts within the nucleus. These findings extend to the realm of glioma stem cells (GSCs), where a physiological state mirroring HS stress inhibits T343 autophosphorylation, thereby inducing the formation of CLK2 condensates and subsequent IR. Notably, expressing the CLK2 condensates hampers the maintenance of GSCs. In conclusion, this research unveils a mechanism by which IR is propelled by CLK2 condensates, shedding light on its role in coping with cellular stress.

## Graphical abstract

Intron retention (IR) is a lesser‐known form of alternative splicing where introns remain in mature mRNA. This study reveals that cell division cycle (CDC)‐like kinase 2 (CLK2) forms condensates under heat shock, driven by its intrinsically disordered region and regulated by phosphorylation. These condensates reorganize nuclear speckles, recruit splicing factors, and promote IR, impacting glioma stem cells and cellular stress response.

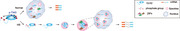

## 1 Introduction

Alternative RNA splicing (AS) is a widespread posttranscriptional process that increases RNA and protein diversity in eukaryotes.^[^ ^1^ ^]^ The main AS types include the cassette‐type alternative exon skipping (SE), alternative 5′ or 3′ splice sites (A5SS or A3SS), mutually exclusive exons (MXE), and intron retention (IR).^[^ ^1^ ^]^ IR involves the inclusion of introns in mature mRNA transcripts and was long considered to be transcriptional noise compared to the other forms of AS.^[^ ^2^ ^]^ While IR has been extensively studied in plants, fungi, insects, and viruses, its role in mammalian cells has only recently been recognized.^[^ ^3^ ^]^

Retaining introns in mRNA can have detrimental effects on protein function, including triggering nonsense‐mediated decay (NMD) or producing truncated proteins.^[^ ^4^, ^5^ ^]^ Alternatively, intron‐retaining mRNAs may be detained in the nucleus, degraded by a mechanism independent of NMD,^[^ ^6^ ^]^ or exported to the cytoplasm upon specific stimuli.^[^ ^7^, ^8^ ^]^ IR regulation has been shown to play critical roles in normal cellular processes such as germ cell differentiation^[^ ^7^ ^]^ and macrophage development and activation,^[^ ^9^ ^]^ as well as various human conditions, such as aging and Alzheimer's disease and cancer.^[^ ^10^, ^11^ ^]^

Nuclear speckles are subnuclear membrane less organelles that are enriched in pre‐mRNA splicing factors (SFs), such as SRSFs and RNAs.^[^ ^12^ ^]^ These speckles are characterized by their dynamic nature and manifest behaviors associated with liquid‐liquid phase separation (LLPS). Within these speckles, constituent RNA‐binding proteins and RNAs continuously undergo exchange with the surrounding nucleoplasm, contributing significantly to gene transcription and splicing.^[^ ^13^ ^]^ The formation of speckles is postulated to arise from multivalent weak interactions between molecules confined within this compartment. Notably, these interactions are often facilitated by the intrinsically disordered region (IDR) present in speckle‐associated proteins.^[^ ^14^ ^]^ However, the role and mechanism of nuclear speckles in regulating specific splicing types, particularly IR, remain elusive.

CLK2 is a member of the Cdc2‐like kinase (CLK) family, which are dual specificity kinases that phosphorylate serine, threonine, and tyrosine residues.^[^ ^15^, ^16^ ^]^ The four CLK members (CLK1, 2, 3, 4) share a similar overall structure and amino acid sequence.^[^ ^17^ ^]^ They are critical splicing kinases that catalyze the phosphorylation of arginine/serine‐rich splicing factors (SRSFs) involved in both constitutive and alternative splicing.^[^ ^18^ ^]^ Phosphorylation of SRSFs by CLKs can stabilize interactions between spliceosome components and promote spliceosome assembly.^[^ ^19^, ^20^ ^]^

In this study, we present an IR regulation mechanism mediated by CLK2 condensates within nuclear speckles. Our investigation demonstrates that HS induces the formation of CLK2 condensates through inhibiting the T343 autophosphorylation. CLK2 condensates exert a regulatory influence on the composition and architecture of speckles, thereby triggering a pervasive occurrence of IR. Our findings provide further insights into the potential role of CLK2 condensates in promoting IR for the maintenance of GSCs, a subset of cells that drives glioma progression.

## 2 Results

### 2.1 HS Induces the Formation of CLK2 Condensates

**Figure 1:**
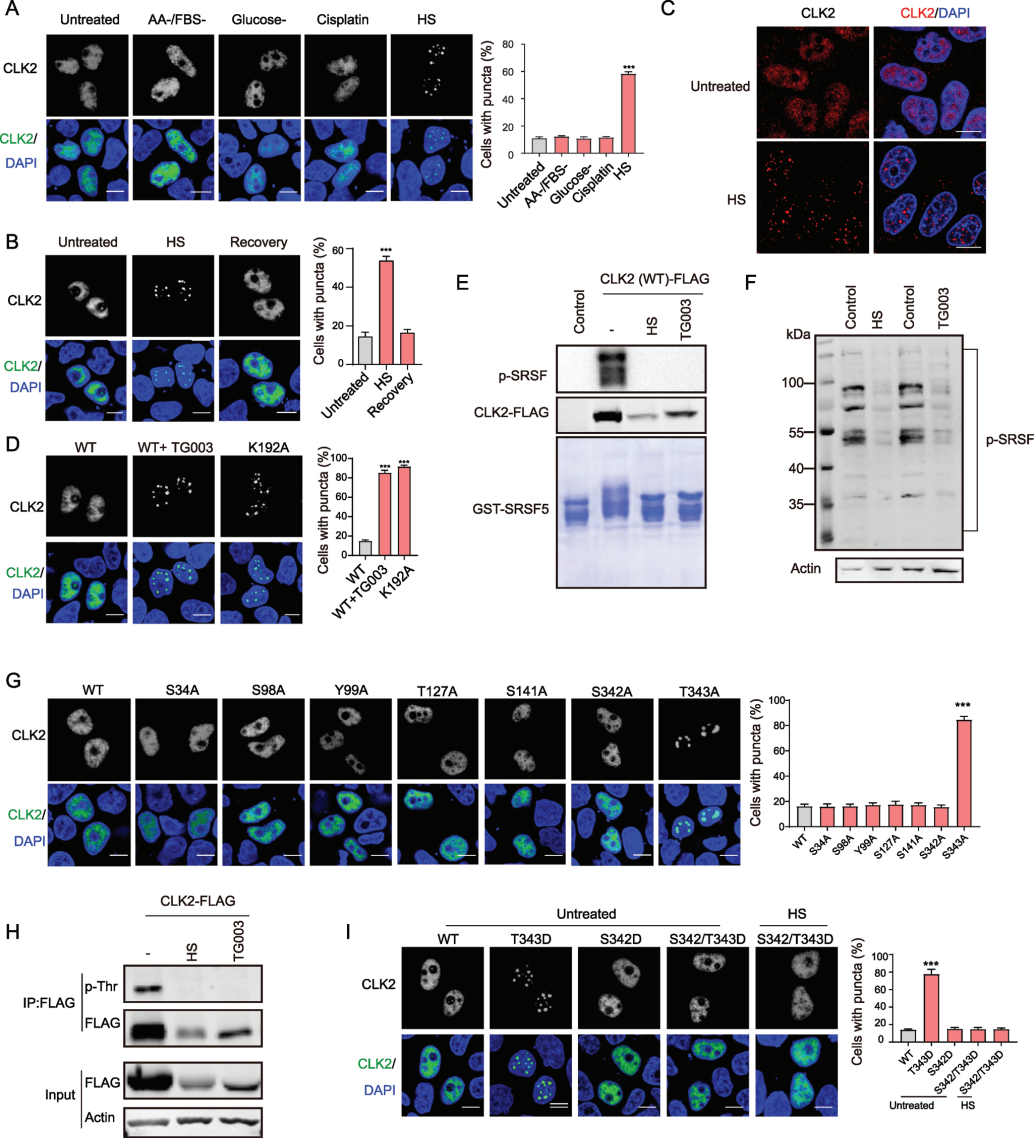
HS induces CLK2 condensate formation via inhibition of T343 autophosphorylation. A) HeLa cells expressing CLK2 (WT)‐GFP‐FLAG were exposed to various stress conditions, including amino acid and FBS deprivation (AA‐/FBS‐, 2 h), glucose withdrawal (6 h), cisplatin treatment (50 × 10^−6^ m, 6 h), or HS (42 °C, 2 h). Subcellular CLK2 protein localization was visualized using a FLAG antibody (green), and nuclei were stained with DAPI (blue). B) HS recovery reverses the CLK2 condensate formation. HeLa cells expressing CLK2‐GFP‐FLAG were subjected to HS (42 °C, 2 h), followed by HS recovery (37 °C, 2 h). C) HS induces the condensate formation of endogenous CLK2 proteins. HeLa cells were subjected to HS (42 °C, 2 h) and stained with the CLK2 antibodies. D) TG003 (10 × 10^−6^ m, 2 h) treatment or K192A mutation induces the formation of CLK2 condensates. (E) HS inhibits CLK2 kinase activity. CLK2 proteins were immunoprecipitated via FLAG‐IP from HeLa cells expressing CLK2 (WT)‐GFP‐FLAG treated with TG003 (10 × 10^−6^ m, 2 h) or subjected to HS (42 °C, 2 h). Kinase activity was assessed using purified GST‐SRSF5 as substrates. FLAG‐IP from the GFP‐expressing cells served as a negative control. Phosphorylation of SRSF5 was detected with a pan‐pSRSF antibody. F) HS inhibits the phosphorylation of SRSF proteins. HeLa cells were treated with TG003 (10 × 10^−6^ m, 2 h) or subjected to HS (42 °C, 2 h). The pan‐pSRSF antibody was used. G) Subcellular localization of the indicated CLK2 mutants. H) HS or TG003 treatment inhibits CLK2 phosphorylation. HeLa cells expressing CLK2 (WT)‐GFP‐FLAG were treated with TG003 (10 × 10^−6^ m, 2 h) or subjected to HS (42 °C, 2 h). CLK2 proteins were immunoprecipitated through FLAG‐IP and their phosphorylation was detected with a phospho‐threonine (p‐Thr) antibody. I) S342/T343D mutation prevented the puncta localization during HS. HeLa cells expressing the indicated CLK2 mutants were cultured in normal growth condition (Normal) or subjected to HS (42 °C, 2 h). A–D,G,I) The percentage of cells displaying nuclear CLK2 puncta was quantified, and the data are presented as the mean ± SD from three independent experiments. ***, *p* < 0.001 (unpaired two‐tailed Student's t test). Scale bars, 10 µm.

As alternative splicing and cellular stresses are closely linked,^[^ ^21^ ^]^ we investigated the localization of CLK2 under various stresses. We found that exposure to HS induced the formation of nuclear puncta (**Figure** **1**A). Other stresses such as amino acid and FBS starvation, glucose depletion, or cisplatin‐induced DNA damage triggered the nuclear translocation of the transcription factor TFEB (Figure S1A, Supporting Information), as previously described.^[^ ^22^, ^23^, ^24^ ^]^ However, they did not change the CLK2 localization (Figure 1A). Notably the punctate localization pattern of CLK2 under HS‐induced stress was reversible upon stress removal (Figure 1B). We conducted analyses using a CLK2 antibody to monitor endogenous CLK2 distribution. Similar to exogenous CLK2, our observations revealed that endogenous CLK2 proteins were mainly diffused within the nucleus and formed nuclear puncta in response to HS (Figure 1C).

### 2.2 T343 Is a Critical Autophosphorylation Residue for Inhibiting the CLK2 Condensate Formation

The formation of CLK2 condensates was also observed in cells treated with TG003, a potent and ATP‐competitive CLK protein inhibitor^[^ ^25^ ^]^ or expressing the kinase‐dead mutant of CLK2 (K192A), in which the ATP‐binding site has been mutated^[^ ^26^, ^27^ ^]^ (Figure 1D). Moreover, the kinase activity of CLKs is sensitive to temperature changes.^[^ ^28^ ^]^ It is conceivable that HS suppresses the kinase activity and thereby induces the formation of CLK2 condensates. To validate the hypothesis, we immunoprecipitated CLK2 and examined the kinase activity toward its substrate SRSF5.^[^ ^26^ ^]^ We observed that CLK2 failed to phosphorylate SRSF5 under HS, as detected by a pan phospho‐SRSF (p‐SRSF) antibody, similar to the effect seen with TG003 treatment (Figure 1E). The presence of multiple bands detected by the pan‐pSRSF antibody may arise from inadequate phosphorylation at various serine sites within the RS domain by CLK2.^[^ ^29^ ^]^ This is evidenced by the disappearance of these p‐SRSF bands as well as the slower migrating bands on Coomassie blue gel following lambda protein phosphatase (λ‐PP) treatment (Figure S1B, Supporting Information). Furthermore, we found that these treatments resulted in severe impairment of intracellular SRSF phosphorylation (Figure 1F). Thus, HS inhibited the kinase activity of CLK2.

The impaired kinase activity may suppress the autophosphorylation of CLK2.^[^ ^30^ ^]^ To uncover the critical phosphorylation site, we mutated each of the known phosphorylation residues (S34, S98, Y99, T127, S141, S342, and T343) within CLK2 proteins^[^ ^27^, ^30^, ^31^ ^]^ to alanine, followed by an evaluation of their subcellular localization. Notably, the T343A mutation prompted the formation of nuclear puncta, whereas the remaining six mutants exhibited predominantly diffuse nuclear distribution (Figure 1G). To detect the T343 phosphorylation in response to HS, we immunoprecipitated CLK2 and examine the phosphorylation through a pan phospho‐threonine (p‐Thr) antibody. We observed a loss of threonine phosphorylation in CLK2 proteins upon HS, or TG003 treatment (Figure 1H).

To further explore the role of T343 phosphorylation, we mutated T343 to aspartate (D) to mimic Thr‐phosphorylation. However, T343D exhibited similar puncta localization to T343A (Figure 1I), indicating its failure to replicate the effects of true phosphorylation, as observed in other studies.^[^ ^32^, ^33^, ^34^ ^]^ Studies have proposed that a pair of vicinal aspartate residues might serve as a more effective phosphomimetic mutation due to the creation of a local double negative charge.^[^ ^35^ ^]^ Thus, we engineered a mutation in both T343 and the adjacent S342, mutated to aspartate (S342/T343D). While this mutant, like the WT, was uniformly localized in the nucleus under normal growth conditions, it failed to exhibit punctate localization under HS (Figure 1I). These findings suggest that the diminished kinase activity of CLK2 hinders T343 autophosphorylation, thus promoting the formation of CLK2 condensates during HS.

### 2.3 CLK2 Undergoes an IDR Dependent LLPS during HS

**Figure 2:**
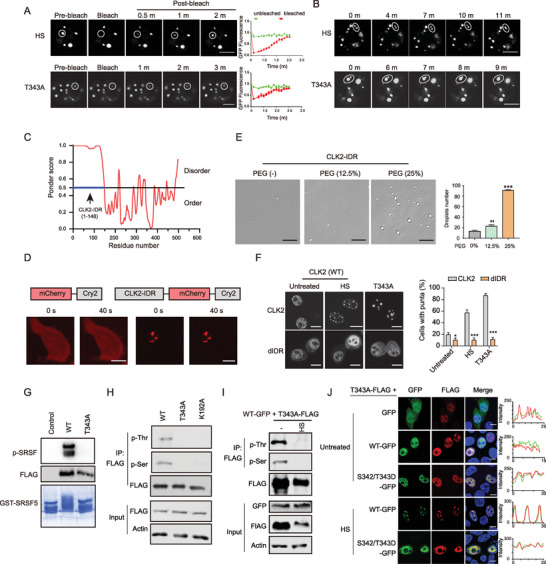
CLK2 condensates exhibit liquid‐like features. A) Representative images of the FRAP experiment involving CLK2 condensates. HeLa cells expressing CLK2 (WT)‐GFP‐FLAG subjected to HS (42 °C, 2 h), while the T343A‐expressing cells remain untreated. Data are presented as the mean ± SD (*n* = 5 droplets). Scale bars, 5 µm. B) Fusion of two CLK2 condensates in the aforementioned cells in (A). Scale bars, 5 µm. C) The intrinsic disorder arrangement of CLK2. The y‐axis displays the PONDER VSL2 score (Ponder score), and the x‐axis represents the amino acid position. The IDR (1‐148 amino acids) within CLK2 (CLK2‐IDR) is indicated by a blue line. D) CLK2‐IDR exhibited phase separation in OptoDroplet assay. CLK2‐IDR was fused with mCherry and Cry2. The constructs were transfected into HeLa cells, and images were captured at the indicated time after stimulation. Scale bars, 10 µm. E) The CLK2‐IDR droplet formation (10 × 10^−6^ m) in a buffer in the absence or presence of PEG6000 (PEG). Scale bars, 10 µm. The droplet number was quantified. Data are presented as the mean ± SD from three independent experiments. **, *p* < 0.01; ***, *p* < 0.001 (unpaired two‐tailed Student's t test). F) IDR deletion inhibits the formation of CLK2 condensates. The dIDR constructs were transfected into HeLa cells. Cells were subjected to HS (42 °C, 2 h). The percentages of cells expressing nuclear CLK2 puncta were quantified. Data are presented as the mean ± SD from three independent experiments. **, *p <* 0.01; ***, *p <* 0.001 (unpaired two‐tailed Student's t test). G) T343A mutation inhibited the kinase activity of CLK2. CLK2 (T343A) proteins were immunoprecipitated, and their kinase activity was assessed. H) CLK2 (T343A) or (K192A) proteins were immunoprecipitated with FLAG antibodies from the respective overexpressing HeLa cells. The immunoprecipitates were detected using a pan‐phospho Thr or Ser antibody. I) CLK2 (WT) phosphorylated the CLK2 (T343A) mutant. HeLa cells were cotransfected with CLK2 (T343A)‐FLAG and GFP or CLK2 (WT)‐GFP. CLK2 (T343A) proteins were immunoprecipitated using a FLAG antibody, followed by phosphorylation detection with a pan‐phospho Thr or Ser antibody. J) HeLa cells were cotransfected with the indicated GFP and FLAG expression constructs and immunostained with FLAG and GFP antibodies. Scale bars, 10 µm.

While LLPS condensates, distinct from protein aggregates, display fluidic properties characterized by marked dynamic behavior,^[^ ^36^ ^]^ we employed photobleaching and time‐lapse imaging experiments to examine the features of CLK2 condensates induced by HS or T343A mutation. Our photobleaching analyses demonstrated that these CLK2 condensates exhibited rapid fluorescence recovery within minutes (**Figure** **2**A). Time‐lapse imaging further revealed the ability to mobilize and fuse spontaneously upon contact (Figure 2B). These findings demonstrated that the CLK2 condensates displayed the LLPS properties.

Given CLK2 contains a predicted IDR (CLK2‐IDR) consisting of the N‐terminal 1–148 amino acids (Figure 2C), we investigated the role of IDR in LLPS through the optoDroplets assay.^[^ ^37^ ^]^ In this assay, we fused the CLK2‐IDR in‐frame to the photoactivatable Cry2 protein. Blue light stimulation induced self‐association of the Cry2 protein, resulting in punctate formation if the IDR was capable of LLPS. We observed that the CLK2‐IDR, but not control proteins, formed droplets even before light exposure (Figure 2D). Furthermore, we conducted an in vitro droplet formation assay with recombinant CLK2‐IDR proteins (Figure S2A, Supporting Information). At a concentration of 10 × 10^−6^ m, CLK2‐IDR formed spherical droplets (Figure 2E). The droplet number increased with an addition of PEG‐6000, a chemical that mimics the crowded cellular environment (Figure 2E) or a high concentration of CLK2‐IDR proteins (Figure S2B, Supporting Information).

We observed that the droplet formation was highly sensitive to increased NaCl concentration (Figure S2C, Supporting Information), suggesting the involvement of electrostatic interactions in mediating the LLPS. We prepared the CLK2‐IDR peptide solutions with or poly(A) or transfer RNA (tRNA). We observed that CLK2‐IDR formed spherical droplets at lower RNA concentrations, while the droplet formation was inhibited at higher RNA concentrations (Figure S2D,E, Supporting Information). These results align with previous findings that lower RNA concentrations enhance electrostatic interactions and promote LLPS, whereas higher concentrations disrupt these interactions, reducing the propensity for condensation.^[^ ^38^, ^39^, ^40^ ^]^

To determine the role of IDR in the context of CLK2 proteins, we constructed IDR deletion (dIDR) mutants. We observed that the absence of IDR failed to induce the formation of condensates in the HS‐stressed CLK2 (WT)‐expressing cells or in the T343A‐expressing cells (Figure 2F). These results collectively indicate the capability and requirement of the IDR for LLPS of CLK2 condensates.

T343 localizes to the activation loop of the CLK2, phosphorylation of which has been suggested to promote the kinase activity.^[^ ^41^ ^]^ Accordingly, the T343A mutant exhibits a loss of kinase activity (Figure 2G). The absence of kinase activity in the T343A mutant likely prevents autophosphorylation at other sites. Consistent with this, we observed the loss of phosphorylation not only at the threonine but also serine residues within the T343A mutant (Figure 2H). When we cotransfected cells with CLK2 (WT) and T343A, we observed that CLK2 (WT) enhanced phosphorylation and prevented the puncta localization of T343A (Figure 2I,J). These effects were abolished in cells subjected to HS (Figure 2I,J), a condition that inhibits the kinase activity of CLK2 (WT), as we have demonstrated in Figure 1E. Unlike the effect of CLK2 (WT), we observed that the phosphomimetic CLK2 (S342/T343D) prevented the puncta localization of T343A under both normal growth and HS conditions (Figure 2J). These results suggest that T343 phosphorylation sustains CLK2 kinase activity and promotes overall autophosphorylation, which inhibits the LLPS activity of the IDR.

### 2.4 CLK2 Condensates Promote IR

**Figure 3:**
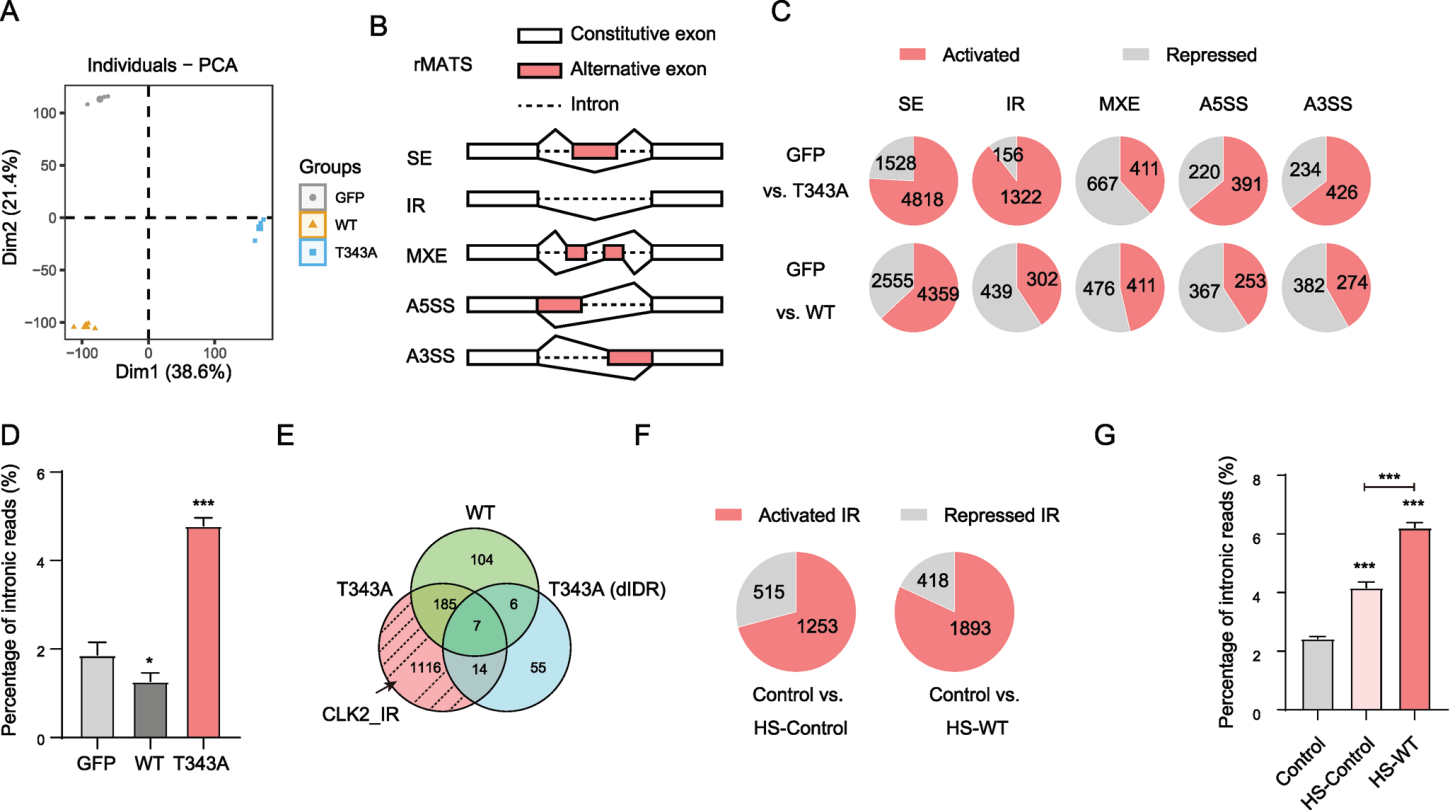
CLK2 condensates induce IR. A) PCA analysis of RNA‐seq data from HeLa cells expressing GFP, CLK2 (WT), and T343A. B) Schematic illustration of the five distinct alternative splicing (AS) types detected by rMATS. C) Number of repressed and activated AS events detected in CLK2 (WT)‐ and T343A‐expressing HeLa cells, in comparison to GFP‐expressing HeLa cells. D) Percentage of RNA‐seq reads mapping to introns for the cells in (A). Data are represented as the mean ± SD from three replicates. *, *p* < 0.05; ***, *p* < 0.001 (unpaired two‐tailed Student's t test). E) Venn diagram depicting the overlap of activated IR events identified in HeLa cells expressing CLK2 (WT), T343A, and T343A (dIDR). A total of 1116 IR events specific to CLK2 condensates (CLK2_IR) is indicated. F) Number of repressed and activated IR events detected in HS‐Control and HS‐WT HeLa cells, compared to HeLa Control cells. G) Percentage of RNA‐seq reads mapping to introns for the indicated HeLa cells. Data are presented as the mean ± SD from three replicates. ***, *p <* 0.001; ns, not significant (unpaired two‐tailed Student's t test).

To investigate the cellular role of CLK2 condensates, we performed high‐throughput RNA‐seq to determine the transcriptome of cells expressing control, CLK2 (WT) or T343A. Through Principal Component Analysis (PCA), a distinct segregation among the three cell groups was discerned (**Figure** **3**A). To investigate the role in alternative splicing, we employed rMATS, an alternative splicing mapping algorithm,^[^ ^42^ ^]^ to analyze the splicing events corresponding to distinct alternative splicing (AS) types in the CLK2 (WT)‐, or T343A‐expressing cells, compared to the control cells (Figure 3B).

Our rMATS analysis revealed a pronounced increase (≈8.5 folds) in the number of activated IR events in the T343A‐expressing cells, while the number of events for other AS types changed to a lesser extent (Figure 3C). We analyzed the RNA‐seq reads that mapped to the genomic regions through read_distribution.py module within the RSeQC package.^[^ ^43^ ^]^ Consistent with the increase of IR events, we observed that the T343A‐expressing cells exhibited an approximately 2‐fold increase in the proportion of RNA‐seq reads mapping to intronic regions in comparison to the other two groups (Figure 3D). We did not observe the increase in the number of activated IR events or RNA‐seq reads mapping to intronic regions or in the CLK2 (WT)‐ or T343A (dIDR)‐expressing cells (Figure 3C,D, Figure S3A–C, Supporting Information). These findings demonstrated that the IDR‐dependent CLK2 condensates promote IR.

The rMATS analysis revealed distinct numbers of activated IR events among the CLK2 (WT)‐, T343A‐, and T343A (dIDR)‐expressing cells. Out of the 1322 activated IR events observed in T343A‐expressing cells, 206 events were also activated in CLK2 (WT) and/or T343A (dIDR) expressing cells (Figure 3E), suggesting that they were not influenced by T343A expression. By excluding these 206 events, we identified 1116 events in the T343A‐expressing cells, which we categorized as the activated IR events specific to CLK2 condensates (CLK2_IR) (Figure 3E, Table S1, Supporting Information).

To determine the role of CLK2 condensates during HS, we analyzed the transcriptome of cells expressing Control, CLK2 (WT) or *CLK2* knockdown (KD) subjected to HS (HS‐Control, HS‐WT and HS‐KD respectively) (Figure S3D–F, Supporting Information). We observed an increase of IR in the HS‐Control cells, as evidenced by a notable increase in the number of activated IR events and the proportion of intronic RNA‐seq reads (Figure 3F,G), consistent with previous findings.^[^ ^21^, ^44^ ^]^ The IR effect was further enhanced by overexpression of CLK2 (WT) upon HS (Figure 3F,G), underscoring the role of CLK2 condensate formation in promoting IR during HS. However, we still observed a general increase in IR in the HS‐KD cells (Figure S3G,H, Supporting Information). This point is discussed further in the discussion section.

To elucidate the activated IR events mediated by CLK2 condensates during HS, we compared the activated IR events in HS‐Control cells (HS‐Control_IR) with CLK2_IR events. Our Venn analysis revealed that 364 of the 1253 HS‐Control_IR events were CLK2_IR events (Figure S3I, Supporting Information). To further investigate whether these 364 events were regulated by CLK2 during HS, we analyzed the activated IR events in the HS‐KD cells (HS‐KD_IR) (Figure S3G, Supporting Information). Among the 364 events, we found that 120 events were not the HS‐KD_IR events (Figure S3I, Supporting Information). The activation of these 244 events in the absence of CLK2 may be attributed to other splicing inhibition mechanisms during HS.^[^ ^21^ ^]^ These findings further suggest that CLK2 condensates promote IR during HS.

### 2.5 Validation of CLK2_IR Events

**Figure 4:**
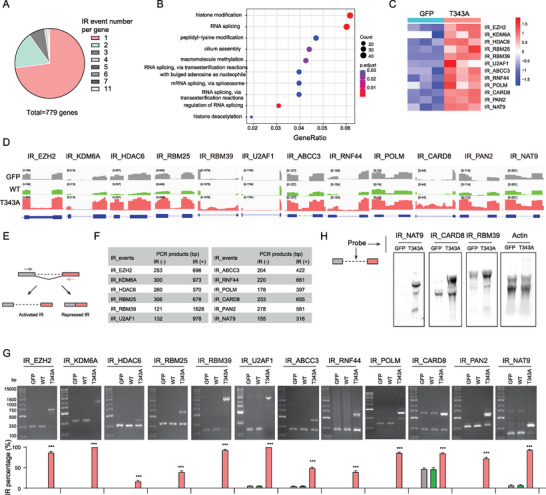
Validation and characterization of CLK2_IR events. A) A pie chart displaying the distribution of genes with varying numbers of CLK2_IR events. B) GO analysis of the 779 genes in (A). Significantly enriched terms are depicted with gene counts and adjusted *p*‐values. C) A heatmap plot showing the IR levels for selected CLK2_IR events in control and T343A‐expressing HeLa cells. D) RNA‐seq read coverage of the indicated gene_IR events in HeLa cells expressing GFP, CLK2 (WT), and T343A. The tracks were group autoscaled and the data ranges were shown. E) Schematic representation of the genomic location of IR validation primers. Introns are depicted with dashed lines, and exons are represented by boxes. Forward and reverse primers are denoted by grey and red arrows, respectively. F) Tables showing the expected sizes of the RT‐PCR products using the primers indicated in (E) for each intron retention (IR) event. IR (+) and IR (‐) denote the RT‐PCR products with and without the intron, respectively. bp, base pair. G) Detection of IR events in HeLa cells expressing GFP, CLK2 (WT), or T343A through RT‐PCR using the IR validation primers. The lower panel displays IR percentages, calculated as the ratio of top binding intensity to the sum of top and bottom binding intensities. *, *p* < 0.05; **, *p* < 0.01; ***, *p* < 0.001 (determined by an unpaired two‐tailed Student's t‐test). H) Detection of transcripts via northern blot, using probes targeting the retained introns for each IR event.

The 1116 CLK2_IR events were found to be distributed among 779 genes and the majority of these genes (72.9%) were affected by only a single IR event (**Figure** **4**A). Notably, these genes were found to be involved in biological processes such as histone modification and RNA splicing, highlighting the significance of these IR events (Figure 4B). Cytoscape analysis identified the top three hub genes associated with CLK2_IR in these processes: EZH2, KDM6A, and HDAC6 for histone modification, and RBM25, RBM39, and U2AF1 for RNA splicing (Figure S4A, Supporting Information). We selected the six CLK2_IR events from these hub genes and an additional six random IR events for RNA‐seq read coverage analysis. Our results showed a significant increase in read counts within the designated intron regions in T343A‐expressing cells, but not in CLK2 (WT) or T343A (dIDR) cells (Figure 4C,D).

To validate these IR events, we conducted RT‐PCR using a flanking primer pair consisting of a forward and reverse primer located in the adjacent upstream and downstream exons of the retained intron, respectively (Figure 4E,F, Table S2, Supporting Information). The RT‐PCR results showed that large amplicons containing the introns were predominantly detected in T343A‐expressing cells (Figure 4G). In contrast, small amplicons lacking the introns were more frequently detected in the control, CLK2 (WT) and T343A (dIDR)‐ expressing cells (Figure 4G and Figure S4B, Supporting Information). To directly assess the presence of IR, we performed northern blot with probes tailored to the retained introns of IR_NAT9, IR_CARD8, and IR_RBM39, respectively. Our findings indicated a significant increase of these intron‐containing transcripts in cells expressing T343A, as detected by the Northern blot signals (Figure 4H). These results validated the occurrence of activated IR events facilitated by the formation of CLK2 condensates.

### 2.6 CLK2 Condensates Reorganize the Speckles

**Figure 5:**
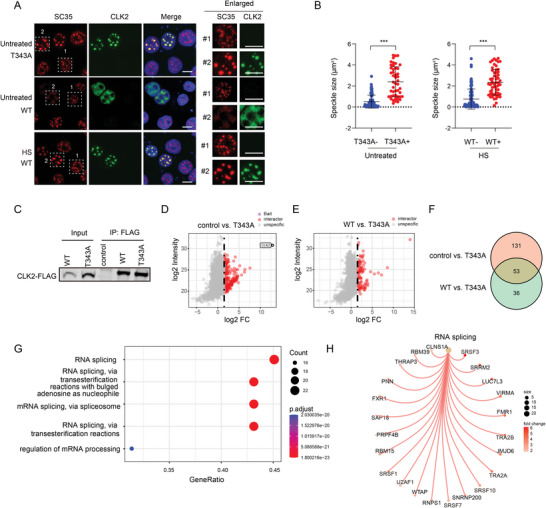
CLK2 condensates reorganize the speckle structure. A) Colocalization of CLK2 condensates with nuclear speckles. HeLa cells were transfected with CLK2 (WT) or T343A‐FLAG expression constructs. The CLK2 (WT)‐expressing cells were subjected to HS (42 °C, 2 h). Cells were costained with FLAG and SC35 antibodies. Higher magnification of the boxed regions (#1 and 2#) is shown on the right panels. Scale bars: 10 µm. B) Quantification of speckle sizes in (A). The speckle sizes in untreated cells expressing T343A (+) or not (‐), as well as in heat shock (HS) stressed cells expressing CLK2 (WT) (+) or not (‐), were determined using ImageJ. Data are presented as the mean ± SD. ***, *p* < 0.001 (unpaired two‐tailed Student's t test). C) Immunoprecipitation (IP) of CLK2 proteins using the FLAG antibody for mass spectrometry analysis. Scale bars: 10 µm. D) Volcano plot illustrating the interacting proteins detected in the T343A‐IP, compared to the control‐IP (control vs T343A). The x‐axis indicates the fold change (FC) of interaction intensity (log_2_ transformed), and the y‐axis indicates the intensity of proteins (log_2_ transformed) detected in the T343A‐IP. The cutoff for log_2_FC is >1.5, as indicated by the dashed line. E) Volcano plot illustrating the interacting proteins detected in the T343A‐IP, compared to the WT‐IP (WT vs T343A). F) Venn diagram displaying the intersection of proteins enriched in the IPs, as shown in (E,F). G) GO analysis of the 53 overlapping genes identified in (G). The top 5 enriched GO terms were shown. Significantly enriched terms are depicted with gene counts and adjusted *p*‐values. H) The gene‐concept network illustrates the connections between the 23 genes and enriched GO terms, specifically focusing on RNA splicing (GO:0016607), as presented in (G). The color intensity of each gene dot corresponds to the fold change (FC) of interaction intensity (log2 transformed) with CLK2 (T343A) proteins. The network visualization was generated using the cnetplot() function included in the enrichplot R package.

Previous studies have shown that the kinase dead mutants of CLKs co‐localize with speckles.^[^ ^26^, ^27^ ^]^ To visualize speckles, we used two antibodies: SC35 antibody, a monoclonal antibody raised against a spliceosomal extract,^[^ ^45^ ^]^ and NREBP antibody that recognizes speckle SON proteins. Our results indicate that the CLK2 condensates induced either by HS or T343A mutation co‐localized with speckles as stained with either SC35 (**Figure** **5**A,B) or NREBP antibody (Figure S5A, Supporting Information).

Nuclear speckles are known to appear as irregular, punctate structures that vary in size and shape.^[^ ^12^ ^]^ We compared the nuclear structures of cells with or without CLK2 expression within the same microscopy field. Interestingly, we observed that the irregular speckle structure was altered to a regular, enlarged, and rounded pattern in untreated cells expressing CLK2 (T343A) or in HS‐stressed cells expressing CLK2 (WT) (Figure 5A,B), similar to those observed when splicing or transcription is inhibited.^[^ ^46^, ^47^ ^]^ In contrast, nuclear speckle formation was inhibited in untreated cells expressing CLK2 (WT) (Figure 5A). Additionally, expression of CLK2‐IDR in the untreated cells resulted in a similar effect on nuclear speckle structures as CLK2 (T343A) (Figure S5B, Supporting Information). These experiments were carried out using HeLa cells, and we observed a similar reorganized speckle structure in T343A‐expressing U251 cells (Figure S5C, Supporting Information). These results suggested that CLK2 condensates may modulate the structure and composition of speckles to promote the IR.

### 2.7 CLK2 Condensates Sequester SFs into Nuclear Speckles

We employed mass spectrometry for the identification of proteins specifically associated with CLK2 condensates (Figure 5C). Our analysis unveiled the enrichment of 184 proteins within the T343A immunoprecipitates (IPs) (Figure 5D, Table S, Supporting Information). To exclude proteins that interacted with CLK2 (WT), we compared the enriched proteins in CLK2 (WT) and T343A IPs and identified 89 proteins present in the T343A‐ but not CLK2 (WT)‐IPs (Figure 5E, Table S3, Supporting Information). The overlap between the initially identified 184 proteins and the subsequent 89 proteins resulted in the identification of a total of 53 proteins that preferentially interacted with T343A (Figure 5F, Table S3, Supporting Information). Subsequent GO analysis elucidated that these 53 proteins primarily participate in RNA splicing processes (Figure 5G). Significantly, 23 out of the 53 proteins exhibited an overlap with the established SFs (Figure 5H).

**Figure 6:**
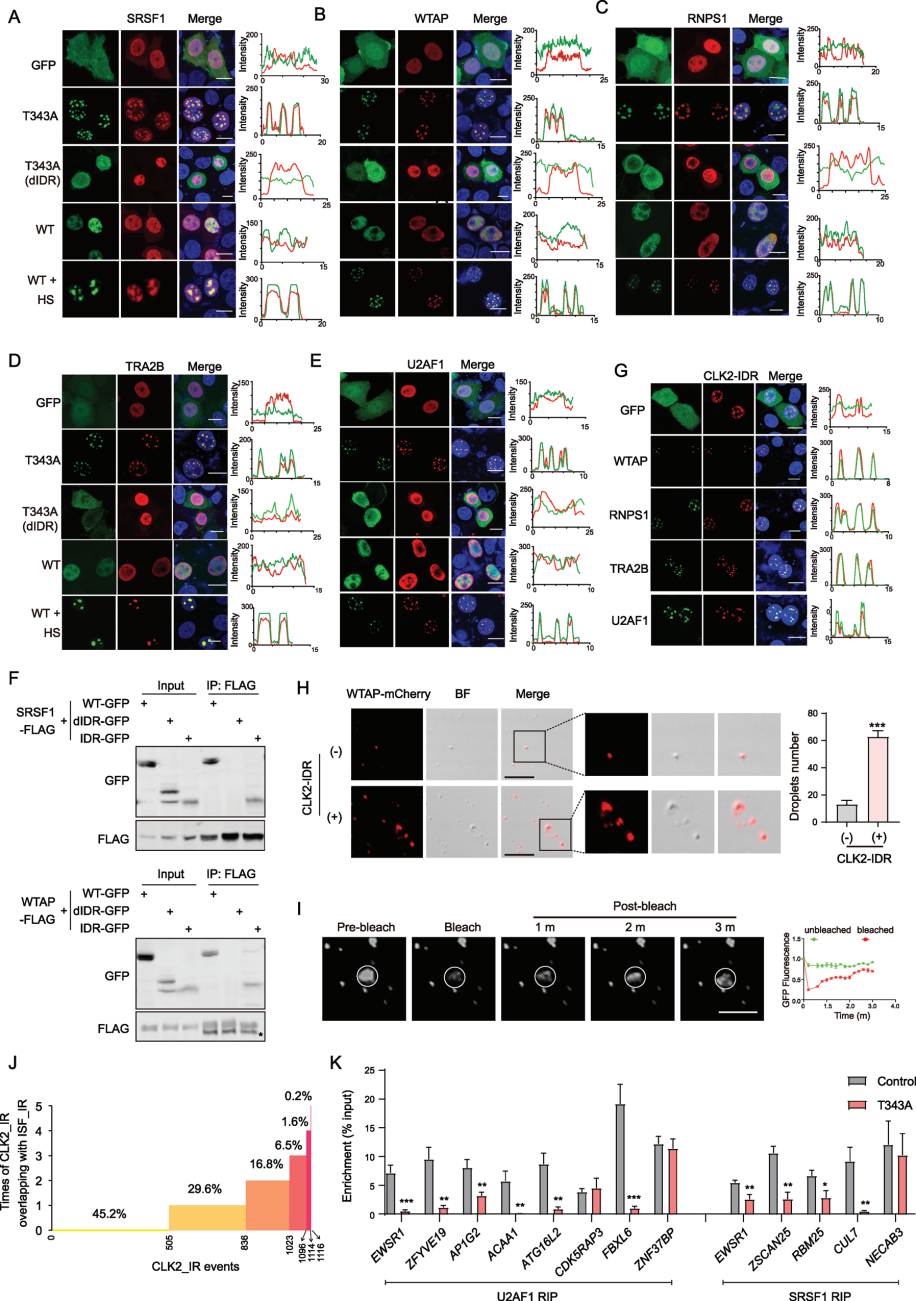
CLK2 condensates sequester SFs within nuclear speckles during HS. A–E) CLK2 condensates recruit SRSF1, WTAP, RNPS1, TRA2B, and U2AF1 to nuclear speckles. HeLa cells were cotransfected with the CLK2‐GFP construct and the construct expressing each of the SFs (FLAG tag). HS (42 °C, 2 h) was applied to the CLK2 (WT)‐expressing cells, while the T343A‐ or T343A (dIDR)‐expressing cells remained untreated. Cells were costained with FLAG and GFP antibodies. The green and red lines in the graphs represent the fluorescence intensity for CLK2 or GFP and SFs, respectively, along the freely positioned arrow. Scale bars, 10 µm. F) CLK2 (T343A) interacted with SRSF1 and WTAP through the IDR. HeLa cells were cotransfected with the indicated constructs and immunoprecipitated with FLAG antibodies. G) Expression of IDR‐mCherry‐CRY2 recruits WTAP, RNPS1, TRA2B, and U2AF1 to the condensates. HeLa cells were cotransfected with the IDR‐mCherry‐CRY2 construct and the construct expressing each of the SFs (FLAG tag). Scale bars, 10 µm. H) CLK2‐IDR promotes the droplet formation of WTAP‐mCherry proteins. The WTAP‐mCherry proteins were incubated with or without CLK2‐IDR proteins in the presence of 10% PEG6000. Scale bars, 10 µm. The droplet number was quantified. Data are presented as the mean ± SD from three independent experiments. **, *p* < 0.01; ***, *p* < 0.001 (unpaired two‐tailed Student's t test). scale bars, 10 µm. I) FRAP analysis of the WTAP‐mCherry proteins incubated with CLK2‐IDR proteins in the presence of 10% PEG6000. Data are presented as the mean ± SD (*n* = 5 droplets). scale bars, 5 µm. J) CLK2_IR events were intersected with activated IR events in cells subjected to knockdown of *CLNS1A*, *FMR1*, *FXR1*, *RBM15*, *RBM39*, *SRSF1*, *SRSF3*, *SRSF7*, *TRA2A*, or *U2AF1* (SF_IR). The y‐axis value indicated the total overlapping times of each CLK2_IR event, while the x‐axis value indicated the number of CLK2_IR events. K) HeLa cells (Control vs T343A) coexpressing U2AF1‐FLAG or SRSF1‐FLAG were immunoprecipitated with FLAG antibodies. The associated RNAs were then analyzed for binding targets using qPCR. Data are presented as the mean ± SD. ***, *p* < 0.001 (unpaired two‐tailed Student's t test).

The 23 SFs play crucial roles in both constitutive and alternative splicing processes, such as the SRSFs (SRSF1, 3, 7, 10),^[^ ^48^ ^]^ TRA2B, and U2AF1. Besides, specific SFs like WTAP and RNPS1 are implicated in the regulation of N6‐methyladenosine methylation of RNAs essential for splicing, as well as mRNA nuclear export and surveillance, respectively.^[^ ^49^, ^50^ ^]^ When expressed with control GFP or CLK2 (WT) proteins, SRSF1 (**Figure** **6**A), WTAP (Figure 6B), RNPS1 (Figure 6C), TRA2B (Figure 6D), or U2AF1 (Figure 6E), was mainly distributed diffusely in the nucleus. However, in cells co‐expressing these SFs with T343A, or when co‐expressed with CLK2 (WT) and subjected to HS, all of these SFs were recruited to the CLK2 condensates (Figure 6A–E). Importantly, the localization of these SFs remained unaltered in cells expressing T343A (dIDR) (Figure 6A–E). Our coimmunoprecipitation assays revealed that T343A and the IDR interacted with SRSF1 and WTAP, while deletion of the IDR prevented T343A from interacting with these SFs (Figure 6F). Additionally, we observed that HS induced an interaction between endogenous CLK2 and SRSF1 proteins (Figure S6A, Supporting Information).

By utilizing the optoDroplets system, wherein CLK2‐IDR is sufficient to induce droplet formation (Figure 2D) and alter the speckle pattern (Figure S5B, Supporting Information), we found that overexpressing CLK2‐IDR‐mCherry‐Cry2 led to continued recruitment of SFs (WTAP, RNPS1, TRA2B, and U2AF1) to speckles (Figure 6G). We purified the WTAP‐mCherry proteins and observed that addition of CLK2‐IDR was capable of promoting the droplet formation of WTAP‐mCherry proteins in the presence of PEG6000 (Figure 6H). Our FRAP analyses demonstrated that these WTAP‐mCherry droplets promoted by CLK2‐IDR exhibited rapid fluorescence recovery (Figure 6I). These results indicated that CLK2 condensates interacted with and recruited these SFs through the IDR.

### 2.8 Sequestration of SFs into the CLK2 Condensates Prevents Their Binding to RNA

Our findings demonstrated that inhibition of the kinase activity induces the condensate formation of CLK2, promoting the IR. Nonetheless, the kinase inhibition may suppress the phosphorylation of SRSFs, resulting in the splicing suppression and promotion of the IR.^[^ ^18^ ^]^ In T343A‐expressing cells exhibiting the increase of IR, however, we did not observe the inhibition of p‐SRSFs (Figure S6B, Supporting Information) and T343A condensates were capable of recruiting p‐SRSFs (Figure S6C, Supporting Information). The presence of CLK2 (WT) or other CLK family members with proficient kinase activity may lead to the phosphorylation of SRSFs,^[^ ^17^ ^]^ as observed in the T343A‐expressing cells. Additionally, CLK2 condensates can recruit SFs (such as WTAP and U2AF1) (Figure 6B–E), which are not phosphorylation substrates of CLK2. These findings suggest that CLK2 condensates are sufficient to induce IR, independently of the phosphorylation status of SRSFs.

Among the 23 SFs that preferentially interacted with CLK2 (T343A), as demonstrated in Figure 5H, 10 have corresponding gene knockdown or knockout RNA‐seq data from HepG2 cells available in the ENCODE database (Table S4, Supporting Information). We analyzed the activated IR events in these SF deficient cells (SF_IR). We found that knockdown of *CLNS1A*, *FXR1*, *RBM39*, *SRSF1*, or *U2AF1* resulted in a greater number of activated IR events than the repressed ones (Figure S6D, Supporting Information), and more than half of the CLK2_IR events (54.8%) were activated under at least one of these knockdown conditions (Figure 6J). Since splicing factors within speckles continuously undergo exchange with the surrounding nucleoplasm to facilitate the splicing process,^[^ ^13^ ^]^ these results suggested that CLK2 condensates may sequester these functional SFs, thereby preventing their access to mRNA.

To address this hypothesis, we investigated whether CLK2 condensates compromised the RNA binding ability of U2AF1 and SRSF1, whose knockdowns resulted in the highest and second‐highest number of activated IR events overlapping with CLK2_IR events, respectively (Figure S6E, Supporting Information). Through analyzing the eCLIP and knockdown RNA‐seq data in the ENCODE database, we identified 30 and 21 binding peaks distributed in 18 and 13 transcripts, for U2AF1 and SRSF1, respectively, overlapped with CLK2_IR regions, as well as the regions for the activated IR events by knockdown of the respective SF (Figure S6F, Supporting Information). Our RIP‐qPCR results validated that U2AF1 and SRSF1 were associated with 8 and 5 of these corresponding transcripts, respectively (Figure S6G, Supporting Information). Notably, T343A expression impaired the binding of U2AF1 and SRSF1 to the majority of these transcripts (75% for U2AF1 and 80% for SRSF1) (Figure 6K). In comparison, we did not observe an association between T343A and the transcripts of which SRSF1 binding was impaired by T343A expression (Figure S6H, Supporting Information), consistent with the fact that CLK2 does not contain any RNA‐binding motifs. These data suggest that the increase in IR may be due to the sequestration of these SFs, thereby preventing their access to mRNAs for splicing activity.

### 2.9 CLK2 Condensates Mediated IR Sequesters Transcripts within the Nucleus and Inhibits Tumor Cell Growth

**Figure 7:**
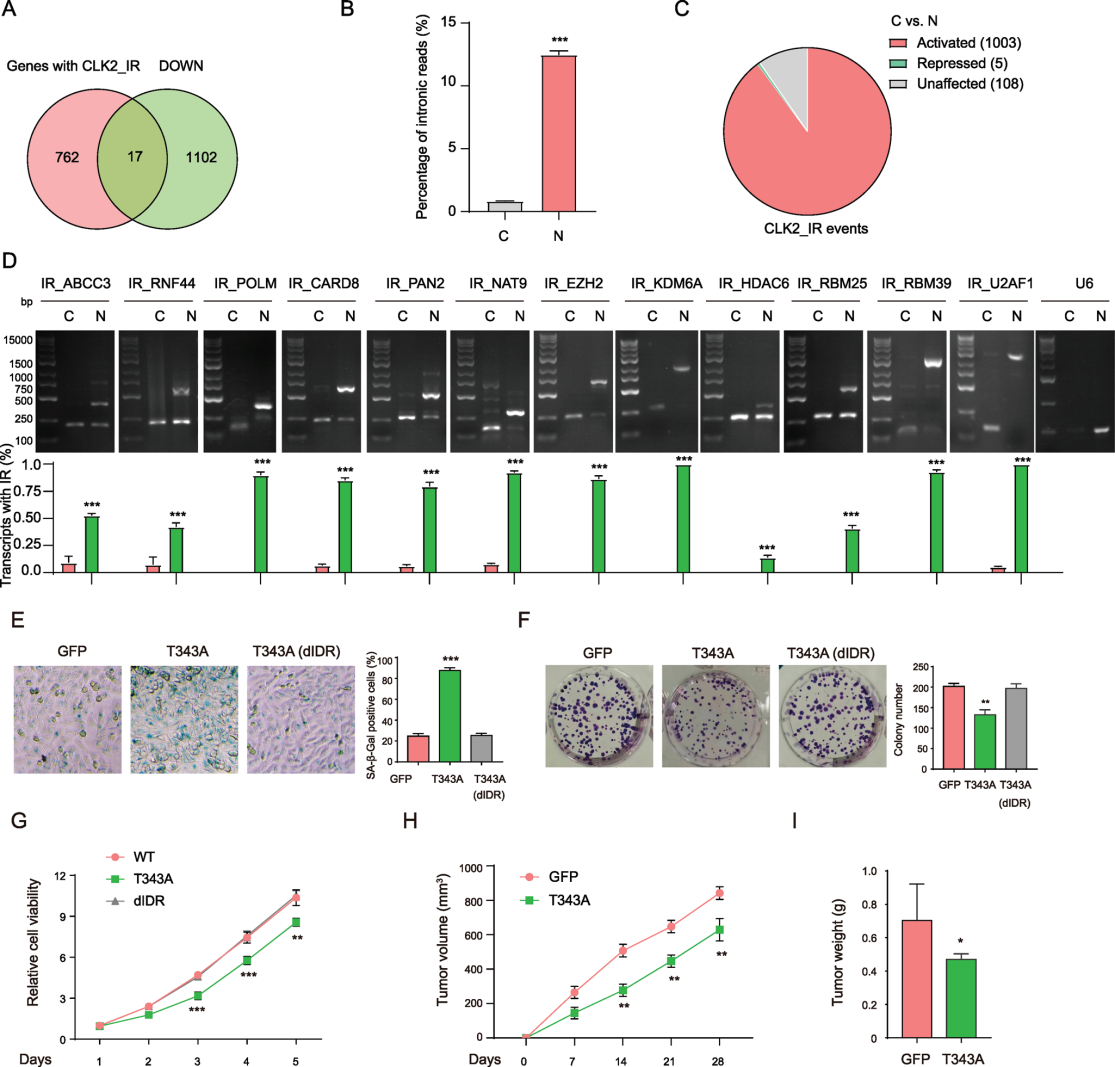
CLK2 condensates mediated IR sequesters the transcripts in the nucleus and suppresses the cell growth. A) Venn diagram illustrating the overlap between the 779 genes mentioned in (A) and the downregulated (DOWN) genes in T343A‐expressing HeLa cells. B) Percentage of RNA‐seq reads mapping to introns for RNAs extracted from the cytoplasmic (*C*) and nuclear (*N*) fractions. Data are represented as the mean ± SD from three replicates. *, *p* < 0.05; ***, *p* < 0.001 (unpaired two‐tailed Student's t test). C) A pie chart illustrating the number of activated, repressed, and unaffected CLK2_IR events in nuclear RNA compared to cytoplasmic RNA (*C* vs *N*). D) RT‐PCR detection of cytoplasmic (*C*) and nuclear (*N*) transcripts in the T343A‐expressing HeLa cells. U6 snRNA detection was used as a control for the nuclear fraction. Data are presented as the mean ± SD from three independent experiments. E) Expression of T343A but not T343A (dIDR) induces cellular senescence in HeLa cells. Senescence was detected by SA‐β‐Gal staining. The bar chart shows the percentage of SA‐β‐Gal‐positive staining cells. Data are presented as the mean ± SD (*n*  =  3) from three independent experiments. F) Expression of T343A but not T343A (dIDR) induces inhibits colony formation in HeLa cells. The bar chart shows the colony number. Data are presented as the mean ± SD (*n*  =  3) from three independent experiments. G) Expression of T343A but not T343A (dIDR) reduces the viability of HeLa cells. Data are presented as the mean ± SD from four replicate wells. (H‐I) T343A expression inhibits tumor growth in a xenograft model. HeLa cells expressing GFP or T343A were subcutaneously injected into nude mice. H) Tumor volume and I) weight are presented as the mean ± SD from 5 mice in each group. D–I) *, *p* < 0.05; **, *p* < 0.01; ***, *p* < 0.001 (unpaired two‐tailed Student's t‐test).

IR can have consequences on the fate of transcripts, such as degradation, nuclear sequestration, or translation into different protein isoforms.^[^ ^2^ ^]^ We found that 97.8% (762 of 779) of these genes, were not downregulated in the T343A‐expressing cells (**Figure** **7**A). We further confirmed that the expression levels of the 12 transcripts, associated with the verified IR events, as depicted in Figure 4, remained unchanged in the T343A‐expressing cells (Figure S7A, Supporting Information). These results suggest that the IR activated by CLK2 condensates does not globally lead to transcript degradation.

As RNA splicing facilitates nuclear export,^[^ ^51^, ^52^, ^53^ ^]^ we examined whether the IR mediated by CLK2 condensates sequestered transcripts in the nucleus. We extracted the cytoplasmic and nuclear RNAs of T343A‐expressing cells for the RNA‐seq analysis. We found that the nuclear RNA exhibited a remarkable increase of intronic reads (Figure 7B). Among the 1116 CLK2_IR events, 1003 events (≈90%) were activated in the nuclear fraction compared to the cytoplasmic fraction (Figure 7C, Figure S7B and Table S5, Supporting Information). We validated that the 12 transcripts associated with the verified CLK2_IR events were predominantly present in the nuclear fraction, whereas transcripts without the IR events were present in both nuclear and cytoplasmic fractions (Figure 7D). These findings demonstrate that the IR activated by CLK2 condensates sequesters transcripts in the nucleus. This nuclear sequestration mechanism, rather than degradation, may enable cells to respond rapidly to heat stress and facilitate the stress recovery.

Sequestration of the transcripts may impair the cellular function of the involved genes and thus the cell growth activity. Our experiments revealed that T343A but not T343A (dIDR) expression induced cellular senescence, as indicated by higher SA‐β‐Gal staining, and inhibited colony formation and cell viability (Figure 7E–G). In a subcutaneous xenograft model, T343A expression significantly reduced both tumor size and weight (Figure 7H–I). Taken together, these results demonstrated that CLK2 condensates inhibit cell growth, likely through the IR and the nuclear sequestration of the transcripts.

### 2.10 The Formation of CLK2 Condensates and IR Is Induced during GSC Differentiation

Recent research advocates for the potential of inducing IR^[^ ^54^, ^55^, ^56^ ^]^ as a treatment strategy for Glioma. GSCs, which represent a subset of cells within glioma, have been shown to play a pivotal role in glioma progression and exhibit significant therapy resistance. IR has been implicated in various stages of development and in cell differentiation.^[^ ^6^, ^7^, ^57^, ^58^, ^59^ ^]^ In light of these, we investigated the significance of our study in GSCs and the differentiated GSCs (dGSCs).

**Figure 8:**
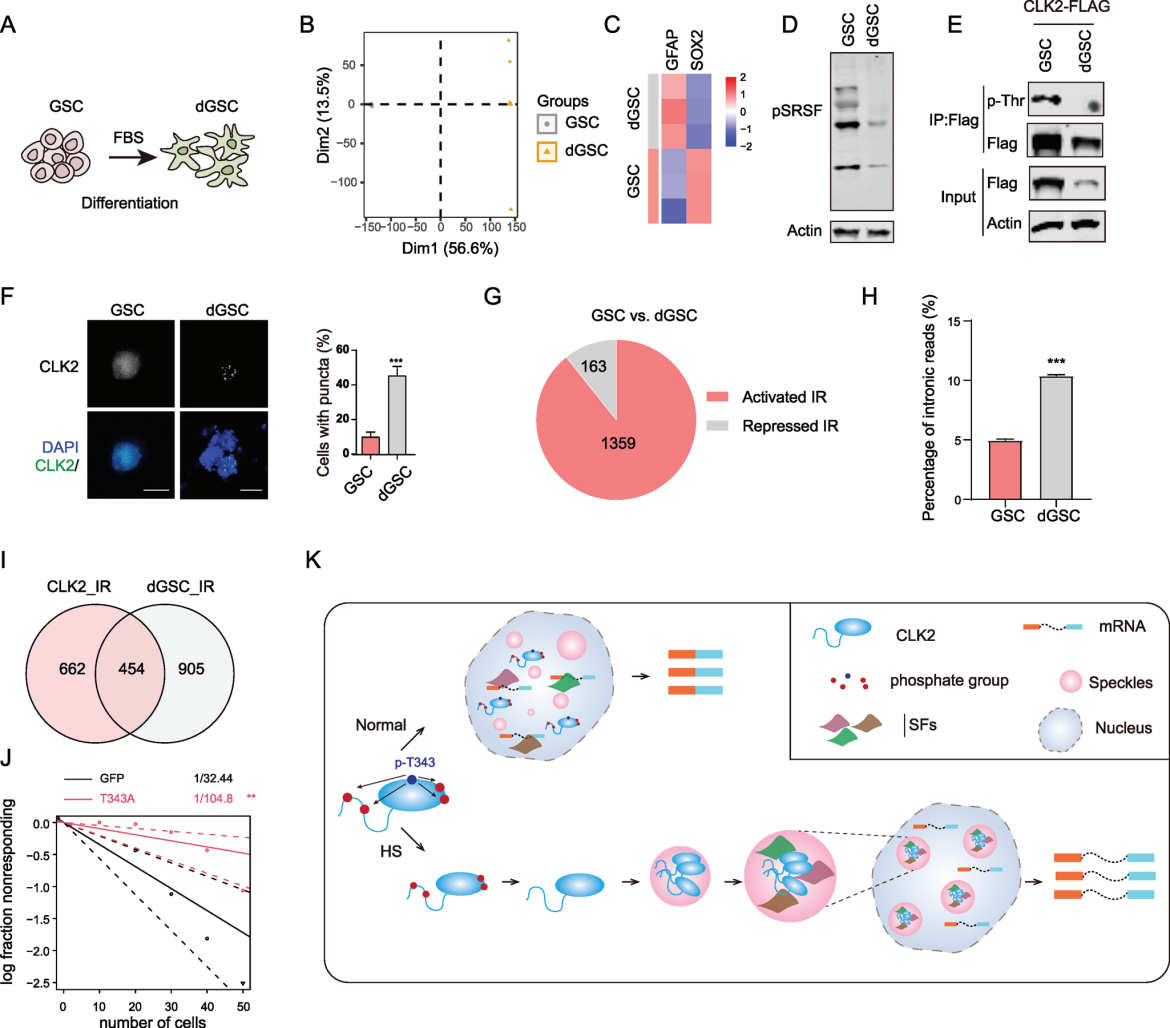
The formation of CLK2 condensates and IR is induced during GSC differentiation. A) A schematic representation of the GSC differentiation model. B) Principal component analysis (PCA) of RNA‐seq data obtained from GSCs and dGSCs. C) A heatmap depicting the expression of GFAP and SOX2 in the GSCs and dGSCs. D) Western blot analysis was conducted using GSC and dGSC lysates. E) CLK2 threonine phosphorylation was suppressed in the dGSCs. CLK2 proteins were immunoprecipitated through FLAG‐IP from GSCs and dGSCs expressing CLK2 (WT)‐GFP‐FLAG, and their phosphorylation was detected with a phospho‐threonine (p‐Thr) antibody. F) Immunofluorescence detection with the Flag antibody was performed on GSCs and dGSCs expressing CLK2‐GFP‐FLAG. Scale bars: 10 µm. G) Number of repressed and activated IR (intron retention) events identified in dGSCs. H) Percentage of RNA‐seq reads mapping to introns for GSCs and dGSCs. Data are presented as the mean ± SD from three replicates. ***, *p* < 0.001 (unpaired two‐tailed Student's t test). I) A Venn diagram illustrates the overlap of CLK2_IR events with the activated IR events in dGSCs (dGSC_IR) and the IR events that negatively correlated with the heat response in GSCs (IR_NC). J) T343A expression inhibits the neurosphere formation of GSCs. GSCs expressing GFP or CLK2 (T343A) were plated at diluted cell numbers, and subsequent neurosphere formation was assessed. ***, *p* < 0.001. The statistics were calculated using extreme limiting dilution analysis.^[^ ^73^ ^]^ K) A model depicting the mechanism and function of IR regulated by CLK2 condensates. Under normal growth conditions, CLK2 undergoes autophosphorylation at multiple residues, with T343 phosphorylation promoting autophosphorylation at other sites. This overall autophosphorylation prevents the formation of nuclear condensates by CLK2. In response to HS, phosphorylation at T343 is inhibited, leading to the loss of autophosphorylation at other residues. The absence of overall autophosphorylation facilitates the formation of CLK2 condensates. These condensates reorganize nuclear speckles into enlarged and rounded structures, recruiting splicing factors (SFs) into the compartment and thereby preventing their binding to mRNAs for intron removal.

We induced the GSC differentiation through serum and subjected the GSCs and differentiated GSCs (dGSCs) to RNA‐seq analysis (**Figure** **8**A,B). Our results unveiled an increase in the expression of GFAP, a marker associated with the astrocytic lineage and a concomitant decline in the expression of SOX2, a GSC marker in the differentiated GSCs (dGSCs) (Figure 8C and Table S6, Supporting Information), indicating the successful initiation of the differentiation process. During the differentiation, we observed a significant reduction in p‐SRSFs and threonine phosphorylation of CLK2, and the emergence of CLK2 condensates (Figure 8D–F). Through analyzing the RNA‐seq data, we found that there was an enhancement of the IR in dGSCs as evidenced by the rMATS analysis and distribution of RNA‐seq reads (Figure 8G,H). These results suggest that the differentiation process mimics the HS stress, leading to the formation of CLK2 condensates and the induction of IR.

We investigated the potential interplay among CLK2 condensates and the activated IR in GSCs. Among the 1116 CLK2_IR events, Venn analysis unveiled that 454 events were activated in dGSCs (Figure 8I and Table S7, Supporting Information). For the six validated CLK2_IR events randomly selected (Figure 4D), three of them (IR_POLM, IR_CARD8, IR_PAN2) were activated in dGSCs through the rMATS analysis (Table S7, Supporting Information). Consistently, a significant increase in read counts within the designated intron regions and an elevation in transcripts containing the introns were observed for these three IR events in dGSCs (Figure S8A,B, Supporting Information). The capability to form neurosphere‐like aggregates has been widely utilized as an in vitro indicator for characterizing the stemness of GSCs.^[^ ^60^ ^]^ We conducted a limiting dilution assay and observed that T343A overexpression robustly impeded neutrosphere formation (Figure 8J). Collectively, these findings unveil a physiological mechanism and role of CLK2 condensates, suggesting that the inhibition of CLK2 condensates suppresses the IR, contributing to the preservation of GSCs.

## 3 Discussion

IR is an important mechanism for cells to cope with various environmental stresses.^[^ ^3^ ^]^ HS, in particular, has been shown to shut down pre‐mRNA splicing, leading to frequent retention of introns in pre‐mRNAs.^[^ ^21^, ^44^ ^]^ Speckles are dynamic structures whose size, shape, and number can vary in response to changing environments.^[^ ^12^ ^]^ They function as expression hubs that supply factors to promote transcription and co‐transcriptional processes, such as splicing.^[^ ^12^, ^13^ ^]^ Our study suggests that nuclear speckles, under the influence of CLK2 condensates, play a role in promoting IR during HS. Mechanistically, CLK2 condensates reorganize speckle structure, resulting in enlarged and rounded speckles and recruit some SFs to the condensates, limiting their access to active transcripts for the intron removal (Figure 8K).

Some SFs recruited by CLK2 condensates, such as SRSFs, were also inhibited through other mechanisms, including dephosphorylation during HS (Figure 1F), which has been shown to inhibit splicing.^[^ ^21^ ^]^ These findings may partially explain the general increase in IR in the HS‐stressed CLK2 KD cells (Figure S3G,H, Supporting Information). Additionally, we observed that HS prompted the punctate formation of other CLK family members (CLK1, CLK3, and CLK4) (Figure S9A, Supporting Information), and the IDRs derived from CLK1, CLK3, and CLK4 were also capable of inducing punctate formation (Figure S9B,C, Supporting Information). Mutation of the equivalent T343 residue induced punctate formation of CLK1 and CLK4 (Figure S9D,E, Supporting Information). These findings suggest that CLK family members share a conserved mode of condensate formation during HS. The redundant role of these members may also contribute to the general increase in IR in the HS‐stressed CLK2 KD cells.

We have demonstrated that CLK2 condensates exhibit characteristics of LLPS, a phenomenon contingent upon the IDR and suppressed by T343 phosphorylation. This observation lends support to the proposition that phosphorylation/dephosphorylation processes regulate the dynamics of speckles.^[^ ^12^ ^]^ Research underscores the significance of charge patterning in facilitating LLPS.^[^ ^39^, ^61^ ^]^ The parameter *κ* was introduced to characterize charge distribution: a *κ* value of 0 represents a uniform alternation of opposite charges along the sequence, while a *κ* value of 1 indicates segregation into two distinct clusters.^[^ ^62^ ^]^ For example, modifying the intrinsically disordered region (IDR) within hTOP1 proteins to a moderately high *κ* value promoted LLPS, likely by enhancing intermolecular electrostatic interactions.^[^ ^39^ ^]^ The CLK2‐IDR contains 48 positively charged residues (44 Arg + 4 Lys) and 20 negatively charged residues (13 Asp + 7 Glu), with a *κ* value of 0.287 calculated using CIDER. This charge distribution likely facilitates the electrostatic interactions that promote the formation of CLK2 condensates. Besides, our results suggested T343 phosphorylation inhibit IDR activity by maintaining the overall phosphorylation status. Such phosphorylation could induce conformational changes that inhibit IDR activity, similar to observations with 4E‐binding protein 2 (4E‐BP2).^[^ ^63^ ^]^ Alternatively, T343 phosphorylation may promote the autophosphorylation within the IDR. The negative charge introduced by phosphorylation within the IDR may intensify electrostatic repulsion, thus impeding phase separation.^[^ ^64^ ^]^ These possibilities require further exploration.

In contrast to the splicing‐suppressing role of CLK2 condensates, the phase separation of other IDR‐containing proteins plays a beneficial role in nuclear speckles and splicing activity. The phase separation of nuclear speckle proteins SRSF1, SRSF2, and U2AF2 involves the incorporation of the hyperphosphorylated RNA polymerase II (Pol II) C‐terminal domain, facilitating the splicing process.^[^ ^65^ ^]^ USP42 condensates orchestrate the integration of the spliceosome component PLRG1 into nuclear speckles, thus enhancing splicing efficiency.^[^ ^66^ ^]^ The SON and SRRM2 proteins are essential for nuclear speckle formation, a function that relies on the extensive IDR regions present in these proteins.^[^ ^67^, ^68^ ^]^ These studies suggest the dual nature of phase separation's impact on speckle activity in splicing.

In addition to speckles, several studies propose that nuclear stress bodies (nSBs), subnuclear organelles formed in response to HS, regulate the IR. A study identified the recruitment of the bromodomain protein BRD4 to nSBs following exposure to HS, resulting in the suppression of IR; however, the underlying mechanism remains unelucidated.^[^ ^69^ ^]^ During the recovery phase after HS, it has been shown that nSBs enhance IR through two distinct mechanisms: phosphorylation of SRSF9^[^ ^70^ ^]^ and sequestration of YTHDC1.^[^ ^71^ ^]^ These findings highlight the varying roles played by nSBs and speckles in the IR regulation during HS and underscore the intricate regulatory network governing IR by a diverse array of molecules within distinct cellular compartments.

TCGA transcriptome analysis of 16 different cancer types indicated a global increase in IR levels in cancerous tissues, compared to the matched normal tissues.^[^ ^11^ ^]^ The increase of IR may contribute to tumor progression by inactivating tumor suppressor genes or generating novel tumor neoepitopes.^[^ ^2^ ^]^ The IR pattern was not examined in glioma likely due to the lack of the matched normal and cancer samples in the TCGA cohorts.^[^ ^11^ ^]^ By utilizing the GSC differentiation model, our findings suggest that the CLK2 condensates‐mediated IR suppresses glioma progression. This implies that IR may exhibit diverse functions in tumor progression contingent upon cancer types. Within glioma, it is well documented that GSCs confer substantial resistance to conventional chemotherapy and radiotherapy.^[^ ^60^, ^72^ ^]^ Our finding provides a means of targeting the GSCs and further supports the potential of inducing IR as a treatment strategy for glioma.^[^ ^54^, ^55^, ^56^ ^]^

## 4 Experimental Section

### Immunofluorescence

Cells grown on cover slides were washed with PBS and fixed with 4% paraformaldehyde for 10 min, followed by permeabilization with 0.5% Triton X‐100 for 5 min. To detect the localization of endogenous CLK2 proteins, cells were subjected to antigen retrieval in a buffer (100 × 10^−3^ m Tris, 5% urea, pH 9.5) (95 °C, 10 min) after the fixation, followed by permeabilization. The cells were blocked with 1% bovine serum albumin (BSA) for 30 min, then incubated with primary antibodies overnight at 4 °C. After washing with PBS, cells were incubated with fluorescent dye‐conjugated secondary antibodies for 1 h. Nuclei were stained with 1 µg mL^−1^ of DAPI. The subcellular localization of proteins was visualized using an Olympus FV3000 laser scanning confocal microscope. The speckle size was determined through ImageJ. Briefly, the image was loaded into the software, and the scale was established using the “Set Scale” tool. Subsequently, the images were converted to 8‐bit, and the threshold was adjusted. Analysis of speckle size was conducted using the “Analyze Particles” tool, whereby the area value of each dot was utilized to indicate the size of individual speckles.

### In Vitro Kinase Assay

HeLa cells were transfected with pHAGE‐CLK2‐GFP‐FLAG expression construct. CLK2 proteins were immunoprecipitated using the FLAG antibody, and then the substrate GST‐SRSF5 was incubated with the immunoprecipitated CLK2 in the kinase reaction buffer [20 × 10^−3^ m HEPES (pH 7.6), 1 × 10^−3^ m MgCl_2_, 0.2 × 10^−3^ m DTT, 150 × 10^−3^ m NaCl, and 1 × 10^−3^ m ATP] for 2 h at 30 °C. After incubation, the reaction was stopped by adding 2x SDS sample buffer, followed by the detection of SRSF5 phosphorylation with a pSRSF antibody. To purify GST‐SRSF5 proteins, SRSF5 cDNA was cloned into pGEX‐6p‐1 with an in‐frame N terminal GST tag and transformed into *Escherichia coli* BL‐21(DE3). GST‐SRSF5 expression was induced with IPTG (0.5 × 10^−3^ m, 30 °C, 4 h) and purified using Glutathione Sepharose 4B (Cytiva, 17075601).

### OptoDroplets Assay

The region for CLK2‐IDR was predicted through PONDR (http://pondr.com/). The CLK2‐IDR was cloned into the optoDroplet plasmids. HeLa cells were transfected with these optoDroplet plasmids. After 24 h, light activation and imaging were performed using an Olympus FV3000 microscope with a 60x oil objective. mCherry positive cells were illuminated by 488 nm light pulses every 2 s and the mCherry fluorescence signal was captured every 2 s.

### Live Imaging and FRAP

HeLa cells expressing the GFP tagged CLK2 proteins were seeded on 35 mm glass‐bottomed dishes (MatTek, P35G‐1.5‐10‐C). FRAP was performed using an Olympus FV3000 microscope with a 488 nm laser. Bleaching was undertaken for 500 ms using 20% laser power (488 nm laser line), and images were collected every 2 s postbleaching. To detect droplet fusion events, live images were acquired using a 60× oil objective with Z and time series. Time lapses were performed at a 35 s interval for 120 cycles.

### In Vitro Droplet Assay

The region of CLK2‐(1‐148) (CLK2‐IDR) was cloned into the pET‐Duet plasmid with an in‐frame His tag and transformed into the Escherichia coli BL‐21(DE3) strain. Expression of His‐CLK2‐IDR was induced with IPTG (0.5 × 10^−3^ m, 30 °C, 4 h) and purified using His60 Ni Resin (Clontech, 635659). The purified CLK2‐IDR proteins were mixed with a droplet formation buffer consisting of 50 × 10^−3^ m Tris‐HCl (pH 7.5), 10% glycerol, and 1 × 10^−3^ m DTT, with or without PEG‐6000. To examine the effect of RNA, CLK2‐IDR was incubated with poly(A) RNA (P9403, Sigma Aldrich) or transfer RNA from baker's yeast (tRNA, AM7119, ThermoFisher Scientific) at various concentrations. The protein solutions were then pipetted onto a glass dish and examined using the Olympus FV3000 laser scanning confocal microscope.

### RNA‐Seq, Differential Gene Expression, Read Distribution, and rMATS Analysis

The RNA library construction and sequencing were performed by Bioyi Biotechnology Co., Ltd. Wuhan, China (details can be found in the Supporting Information). The RNA‐seq raw reads were quality‐controlled and trimmed using Fastp (v0.20.1) with default parameters. The sequencing read information for each sample was shown in Table S8 (Supporting Information). The information includes the number of raw and clean read and total bases, the rate for sequencing quality scores (Q20 and Q30) and GC percentages. The resulting clean reads were aligned to the human reference genome (GRCh38) using STAR (v2.2.1), and BAM files were generated for each sample. The BAM files were sorted and indexed using Samtools (v1.14). The number of reads (counts) per gene was determined using featureCounts (v2.0.1). Genes expressed in at least half of the samples were selected for differential gene expression analysis using the limma (v3.50.3) package with a cutoff of adjusted *p*‐value < 0.05 and | log2FC | ≥ 1. Gene body coverage was evaluated using the read_distribution.py module from the RSeQC python package.^[^ ^43^ ^]^

rMATS (v4.1.2) was used to analyze the BAM files and identify differentially spliced events between the control and experimental groups. rMATS quantified five types of AS events: ES, A5SS, A3SS, MXE, and IR. The IncLevelDifference was calculated as the variance between the mean inclusion levels observed in the control and experimental groups, and used to represent the ΔPSI value (percentage spliced in). AS events with FDR < 0.1 & ΔPSI >0.05 were considered as significantly repressed and AS events with FDR < 0.1 & ΔPSI < (−0.05) as significantly activated in the experimental group. The IncLevel value was used to indicate the IR level and to plot the heatmap. The resulting BAM files were converted to BigWig format using deepTools (v 3.5.1) to reveal the coverage of RNA‐seq reads by Integrative Genomics Viewer (IGV).

### Gene Ontology (GO) Analysis and Identification of Hub Genes

To identify the enriched biological pathways, the genes associated with CLK2_IR events were subjected to GO analysis using the enrichGO() function with default parameters in the clusterProfiler R package (v4.2.2). The CLK2_IR genes involved in histone modification or RNA splicing were used for the protein‐protein interaction (PPI) analysis. The PPI network was derived based on the Search Tool from the Retrieval of Interacting Genes (STRING) database and the interactions were selected with a confidence score > 0.4. The results of this analysis were visualized with Cytoscape (v3.10.1). cytoHubba was used, a plug‐in of Cytoscape, to filter the hub genes from the whole PPI network and calculated it by the maximal clique centrality (MCC) method.

### Cytoplasm and Nuclear RNA Extraction

Cells grown in 10 cm dishes were washed once in PBS and collected with 500 µL of ice‐cold hypotonic buffer (10 × 10^−3^ m HEPES, pH 7.9, 10 × 10^−3^ m KCl, 0.1 × 10^−3^ m EDTA, 0.1 × 10^−3^ m EGTA, 1 × 10^−3^ m DTT, 0.15% NP‐40). The cell lysates were homogenized with a Dounce homogenizer and centrifuged at 12 000 rpm for 5 min. The supernatants were collected as the cytoplasmic fraction, while the pellets were collected as the nuclear fraction. The RNA in each fraction were extracted by Trizol.

### Northern Blot

Total RNA was separated by formaldehyde gel electrophoresis. The negatively charged RNA was transferred onto a nylon membrane overnight by capillary action. The membranes were then cross‐linked to the RNA using a UV‐light cross‐linker (254 nm, 120 mJ cm^−^^2^, 45 s). The probes were labeled with biotin using a random primer DNA labeling kit (Beyotime, China, D3118). The probe sequences were listed in Table S9 (Supporting Information). Subsequently, the RNA fixed on the membrane was hybridized with the biotin‐labeled probe at 68 °C for 16 h. The membranes were incubated with a streptavidin–HRP conjugate, and the hybridization signal was developed using an enhanced chemiluminescence reagent. The blots were then exposed using a chemiluminescent imaging system.

### Immunoprecipitation (IP) and Mass Spectrometry (MS) Analysis

Cells were lysed in IP lysis buffer (20 × 10^−3^ m Tris‐HCl, 100 × 10^−3^ m NaCl, 5 × 10^−3^ m EDTA, 0.2% NP40, 16% glycerol, and protease inhibitors) and subsequently incubated with IP antibodies prebound to Dynabeads protein G (Thermo Fisher Scientific, 10004D). The IP beads underwent three washes with CO‐IP lysis buffer and were then boiled for 5 min in 40 µL of 2× SDS sample buffer (100 × 10^−3^ m Tris‐HCl, pH 6.8, 4% SDS, 20% glycerol, 0.2 m dithiothreitol, and 0.02% bromophenol blue). IP lysates were subjected to MS analysis to identify interacting candidates. Further details regarding the MS protocol can be found at the PRIDE partner repository with the dataset identifier PXD040997.

### RNA Immunoprecipitation (RIP)

HeLa cells (2 × 10⁷) were rinsed with ice‐cold PBS and suspended in 1 mL RIPA buffer supplemented with RiboLock RNase Inhibitor, Protease Inhibitor Cocktail, and Phosphatase Inhibitor Cocktail. Cell lysates were centrifuged at 1000 x *g* for 10 min at 4 °C, and the supernatants were precleared with 10 µL Dynabeads Protein G (Thermo Fisher Scientific, 10004D). The precleared supernatants were then equally divided and incubated with 20 µL Dynabeads Protein G and FLAG or mouse IgG antibodies for 2 h at 4 °C. This was followed by washing three times with NT2 buffer (50 × 10^−3^ m Tris‐HCl pH 7.5, 150 × 10^−3^ m NaCl, 1 × 10^−3^ m MgCl₂, 0.05% NP‐40). The beads were resuspended in Proteinase K buffer, and RNA was extracted. RT‐qPCR was then performed using the primers listed in Table S10 (Supporting Information).

Cell culture, plasmids and reagents, plasmid transfection and viral infection, Western blot, RT‐PCR, SA‐β‐Gal staining, cell viability and subcutaneous xenograft model, and RNA‐seq library construction and sequencing (See the Supporting Information for details).

### Statistical Analysis

All experimental data were examined at least three times. Statistical analysis was performed using GraphPad Prism software or R packages. The statistical tests used, including Student's t test and Wilcox test were indicated in the respective figure legends. A *p*‐value of <0.05 was considered statistically significant, and the significance levels were denoted as follows: *, *p* < 0.05; **, *p* < 0.01; ***, *p* < 0.001.

## Conflict of Interest

The authors declare no conflict of interest.

## Supporting information

Supporting Information

Supplemental Figures 1‐9

Supplemental Tables 1‐10

Supplemental Figure

## Data Availability

RNA‐seq raw data have been deposited in Gene Expression Omnibus under the accession numbers GSE227673, GSE271918, GSE247418, and GSE247419. The mass spectrometry proteomics data have been deposited to the ProteomeXchange Consortium via the PRIDE partner repository with the dataset identifier PXD040997.
