# Supplementary material for: CLK2 Condensates Reorganize Nuclear Speckles and Induce Intron Retention
Source: Adv Sci (Weinh). 2024 Aug 9;11(38):2309588. doi: 10.1002/advs.202309588 (PMC11481226; doi:10.1002/advs.202309588)
Supplement: Supplementary file 1 — Supporting Information [file ADVS-11-2309588-s003.docx]

**Supplemental Materials and Methods**

**Cell culture**

HeLa, U251 and HEK293T cells were obtained from ATCC and cultured in Dulbecco's modified Eagle's medium (DMEM) (Biological Industries, 01-052-1A), supplemented with 10% fetal bovine serum (FBS) (Biological Industries, 04-001-1A), 100 units/mL penicillin, and 100 μg/mL streptomycin (Gibco, 15070063). The GSC387 was kind gifts from Dr. Jeremy N Rich (Department of Neurology, University of Pittsburgh, Pittsburgh, Pennsylvania), Dr. Shideng Bao (Department of Cancer Biology, Lerner Research Institute, Cleveland Clinic, Cleveland, USA), and Dr. Xingjiang Yu (Department of Histology and Embryology, Huazhong University of Science and Technology). GSC387 were cultured in Neurobasal™-A (Gibco 10888022) supplemented with B27 (Gibco 17504-044), 20 ng/ml EGF (PrimeGene 105-04), and 20 ng/ml basic fibroblast growth factor (PrimeGene 104-02), as previously described ^1^. To induce the differentiation, the GSCs were cultured in DMEM growth media containing 10% FBS for 14 days^2^. All cells were incubated at 37°C in a humidified 5% (v:v) CO_2_ incubator.

**Plasmids and reagents**

The coding regions for CLK2 were cloned into the pHAGE expression vector with either an in-frame N-terminal myc tag or a C-terminal FLAG tag. The CLK2 mutants were constructed by PCR-directed mutagenesis using the CLK2 (WT) expression construct as templates. The coding regions for SRSF1, TRA2B, RNPS1, WTAP, or U2AF1 were cloned into the pHAGE expression vector. The shRNA (5’ CTATCGGCATTCCTATGAATA 3’) to knock down CLK2 was cloned into the pLKO.1 vector. The antibodies are as follows: p-SRSF (Sigmaaldrich, MABE50), p-Thr (Cell Signaling Technology, 9386), p-Ser (Santa Cruz, sc-81514), Actin (Sigmaaldrich, A1978), FLAG (Sigmaaldrich, F3165), His (Santa Cruz, sc-53073), GFP (Proteintech, 50430-2-AP), CLK2 (Thermo Fisher Scientific, PA5106489) , SC35 (abcam, ab11826), NREBP (Santa Cruz, sc-398508), SRSF1 (Proteintech, 12929-2-AP), IRDye® 800CW Goat anti-Mouse (LI-COR, 92632210), IRDye® 800CW Goat anti-Rabbit (LI-COR, 92632211), Alexa Fluor™ 488 Goat anti-Mouse IgG (H+L) (Thermo Fisher Scientific, 1939600), and Alexa Fluor™ 568 Goat anti-Rabbit IgG (H+L) (Thermo Fisher Scientific, 1942295). Other reagents are as follows: DAPI (Sigma-Aldrich, #D8417), benzoNuclease (Novoprotein, M056), TG003 (MCE, HY-15338), and puromycin (Sigma-Aldrich, P9620).

**Plasmid transfection and viral infection**

For gene transient expression, plasmids were transfected into cells with a homemade polycation polyethyleneimine (PEI) reagent. To produce lentivirus for target cell infection, pHAGE or pLKO.1 expression constructs were co-transfected with psPAX2 (Addgene, 12,260) and pMD2.G (Addgene, 12,259) into HEK293T cells. Virus in the culture media was filtered through a 0.45 mm filter and used to infect HeLa and U251 cells. Forty-eight hours after infection, cells were selected with puromycin (1 μg/ml). The surviving cell population was examined for the target gene expression.

**Western blot**

Cells were lysed in RIPA buffer (50 mM Tris-HCl pH 7.4, 150 mM NaCl, 1 mM EDTA, 1% Triton X-100, 0.5% sodium deoxycholate, 0.1% SDS) supplemented with protease inhibitor cocktails (Roche, 05892970001). Equal amounts of proteins were separated by SDS-PAGE and transferred to 0.45 µm pore-size nitrocellulose membranes. Membranes were blocked with 5% skim milk for 0.5 hour at room temperature, then incubated with primary antibodies overnight at 4°C. The following day, membranes were incubated with dye 800- or dye 700- conjugated secondary antibodies for 1 hour. Fluorescence signals were captured using a Licor Odyssey-CLx machine.

**RT-PCR**

Total RNA was isolated using TRIzol reagent (Thermo Fisher Scientific, 15596026), and reverse transcription was performed using a cDNA reverse transcription kit (Thermo Scientific, 4374966). Semi-quantitative RT-PCR was carried out using primers listed in Table S5 to detect transcripts with or without IR. The amount of each isoform, as indicated by the intensity of respective PCR products, was quantified using ImageJ.

**SA-β-Gal staining**

Senescence β-Galactosidase Staining Kit (Beyotime Biotechnology, China, C0602) was used for SA-β-Gal staining. Cells (2× 10^4^ per well) were seeded in 12-well plates. After 24 hours, cells were washed with 1× PBS and sequentially incubated with fixation buffer and staining solution mixture. Cells were then incubated at 37°C overnight and the staining signals were observed under an inverted microscope.

**Cell Viability**

Cell viability was determined by CellTiter-Glo® Luminescent Cell Viability Assay according to the manufacturer's instructions (Promega, #G7571). Briefly, cells were seeded in 96-well plates (1× 10^3^ cells per well). At the indicated days, Cell-Titer Glo reagent was added to cells, and the signal was captured using a luminometer. All data were normalized to day 0 to show the relative cell proliferation rates.

**Subcutaneous xenograft model**

All animal experiments were approved by the Ethical Committee of Tongji Medical College, Huazhong University of Science and Technology. BALB/C-nu/nu nude mice (4-week-old, male) were obtained from GemPharmatech and randomly divided into groups of five mice each. Approximately 3 × 10^6^ HeLa cells expressing either GFP or CLK2 (T343A) were injected into the flank of the right arm. Tumor volumes were calculated using the formula: 1/2 (length × width^2^). Mice were euthanized 35 days after the injections, and tumors were extracted and weighed.

**RNA-seq library construction and sequencing**

The RNA library construction and sequencing were performed by Bioyi Biotechnology Co., Ltd. Wuhan, China. In brief, mRNA isolation from total RNA was executed utilizing poly-T oligo-attached magnetic beads. Subsequently, mRNA underwent fragmentation in a buffer containing divalent cations at an elevated temperature. The initial strand of cDNA was synthesized using random hexamers, succeeded by the synthesis of the second cDNA strand. The resultant double-stranded cDNA underwent repair and had an 'A' added to its 3' end. Purification and fragment selection were achieved through Hieff NGS® DNA Selection Beads. Following PCR amplification and enrichment of the products, the target region library underwent denaturation, cycling, and digestion, yielding single-stranded circular DNA. Rolling circle amplification was then employed to amplify the single-stranded circular DNA. The RNA library was sequenced using the MGI T7 platform (BGI Inc., Shenzhen, China), yielding 2 × 150 bp paired-end read datasets.

1 Huang, W. *et al.* A novel EGFR variant EGFRx maintains glioblastoma stem cells through STAT5. *Neuro Oncol*, doi:10.1093/neuonc/noad153 (2023).

2 Zhang, G. *et al.* Chromatin remodeler HELLS maintains glioma stem cells through E2F3 and MYC. *JCI Insight* **4**, doi:10.1172/jci.insight.126140 (2019).

**Supplementary figure legends (Fig. S1-9)**

**Fig. S1 (Related to Fig. 1)**

1. The subcellular localization of TFEB in response to various stresses. HeLa cells expressing TFEB-GFP were subjected to the stresses, as indicated in Fig. 1A, followed by the examination of TFEB-GFP localization. The percentage of cells displaying nuclear CLK2 puncta was quantified, and the data are presented as the mean ± SD from three independent experiments. ***, p < 0.001 (unpaired two-tailed Student’s t test). Scale bars, 10 µm.
2. The pSRSF5 binds disappeared by the lambda protein phosphatase (λ-PP) treatment. CLK2 proteins were immunoprecipitated via FLAG-IP from HeLa cells expressing CLK2 (WT)-GFP-FLAG Kinase activity was assessed using purified GST-SRSF5 as substrates. After the in vitro kinase assay, the mixture was incubated with λ-PP (20 units/µl).

**Fig. S2 (Related to Fig. 2)**

1. The purified His-CLK2-IDR proteins were visualized via Coomassie Blue staining (CB) and Western Blot (WB) analysis using an α-His antibody.
2. Representative images of CLK2-IDR droplet formation in various concentrations.
3. Representative images of CLK2-IDR droplet formation (20 μM) in buffer containing the indicated concentrations of NaCl.
4. Representative images of CLK2-IDR droplet formation (10 μM) in the presence of Poly(A).
5. Representative images of CLK2-IDR droplet formation (10 μM) in the presence of tRNA.

Panel B-E: scale bars, 10 µm; quantification of droplet number is presented in the bar graph; data are presented as the mean ± SD from three independent experiments. *, *p* < 0.05; ***, *p* < 0.001 (unpaired two-tailed Student’s t test).

**Fig. S3 (Related to Fig. 3)**

1. PCA analysis of RNA-seq data from HeLa cells expressing GFP and T343A (dIDR).
2. The number of AS events that were either repressed or activated in T343A (dIDR)-expressing HeLa cells, in comparison to GFP-expressing cells [GFP vs. T343A (dIDR)].
3. Percentage of RNA-seq reads mapping to introns for the cells in panel (A). Data are presented as the mean ± SD from three replicates. ***, *p <* 0.001 (unpaired two-tailed Student’s t test).
4. PCA analysisof RNA-seq data from Control, HS-Control, HS-WT, and HS-KD HeLa cells. The cells were subjected to HS (42 °C, 4 hours).

(E-F) Examination of CLK2 knockdown efficiency. The cells expressing the control (sh-Control) or shRNA targeting CLK2 (sh-CLK2) were collected for RT-qPCR and western blot detection.

1. Number of repressed and activated IR events detected in HS-CLK2 KD HeLa cells, compared to Control HeLa cells.
2. Percentage of RNA-seq reads mapping to introns for the indicated HeLa cells. Data are presented as the mean ± SD from three replicates. ***, *p <* 0.001; *ns*, not significant (unpaired two-tailed Student’s t test).
3. Venn diagram depicting the overlap of CLK2_IR events and the activated IR events identified in HeLa control or CLK2 KD cells subjected to HS (HS-Control_IR, HS-KD_IR).

**Fig. S4 (Related to Fig. 4)**

1. The top three hub genes associated with CLK2_IR in histone modification (left panel) and RNA splicing (right panel).
2. Detection of IR in HeLa cells expressing GFP and T343A (dIDR) through RT-PCR. The lower panel displays IR percentages, calculated as the ratio of top binding intensity to the sum of top and bottom binding intensities. *, p < 0.05; **, p < 0.01; ***, p < 0.001 (determined by an unpaired two-tailed Student’s t-test).

**Fig. S5 (Related to Fig. 5)**

1. Co-localization of CLK2 condensates with nuclear speckles. HeLa cells expressing CLK2 (WT) were subjected to heat shock (HS), while T343A-expressing HeLa cells remained untreated. Cells were co-stained with FLAG and NREBP antibodies. Scale bars, 10 μm.
2. HeLa cells were transfected with the CLK2-IDR-mCherry-CRY2 construct and subsequently immunostained with mCherry and SC-35 or NREBP antibodies. Scale bars: 10 μm.
3. The T343A-expressing U251 cells were stained with FLAG and SC-35 antibodies to visualize the exogeneous CLK2 and nuclear speckles, respectively. Scale bars, 10 μm.

**Fig. S6 (Related to Fig. 6)**

1. HS induced the interaction between endogenous CLK2 and SRSF1 proteins. HeLa cells untreated or subjected to HS (42 °C, 2 hours) were immunoprecipitated with Control (IgG) or SRSF1 antibody, followed by detection with the indicated antibodies.
2. T343A expression does not affect the phosphorylation of SRSFs. Hela cells expressing GFP or T343A-GFP were collected for the western blot detections.
3. T343A recruits phosphorylated SRSFs into the condensates. HeLa cells were transfected with GFP or T343A-GFP constructs. Scale bars, 10 µm.
4. A bar plot showing the number of activated and repressed IR events in the SF knockdown cells.
5. A bar plot showing the number of activated IR events in SF knockdown cells that overlapped with CLK2_IR.
6. Venn diagram displaying the intersection of activated IR events in SRSF1 or U2AF1 knockdown (KD) cells (U2AF1 KD_IR, SRSF1 KD_IR) and CLK2_IR events that have binding peaks for SRSF1 or U2AF1. The number in the bracket indicated the number of transcripts associated with the intersected events.
7. Validation of RNA binding peaks of SRSF1 or U2AF1 through RIP-qPCR. The 18 transcripts for U2AF1 and 13 transcripts for SRSF1, as indicate in panel (D), were examined.
8. CLK2 (T343A) does not bind to the SRSF1 targets. HeLa cells expressing SRSF1-FLAG or CLK2-FLAG were immunoprecipitated with FLAG antibodies. The associated RNAs were then analyzed for binding targets using qPCR. Data are presented as the mean ± SD. ***, p < 0.001 (unpaired two-tailed Student’s t test).

**Fig. S7 (Related to Fig. 7)**

1. RNAs were extracted from HeLa cells expressing GFP or T343A. The expression of transcripts associated with the indicated IR events were examined through qPCR. Data are presented as the mean ± SD.
2. A heatmap plot showing the IR level of the indicated IR events in the nuclear RNAs compared to the cytoplasmic RNAs from T343A-expressing HeLa cells.

**Fig. S8 (Related to Fig. 8)**

1. RNA-seq read coverage of the indicated gene_IR events in the GSCs and dGSCs. The tracks were group autoscaled and the data ranges were shown.
2. Detection of the indicated gene_IR events in GSCs and dGSCs through RT-PCR. The lower panel displays IR percentages, calculated as the ratio of top binding intensity to the sum of top and bottom binding intensities. *, p < 0.05; **, p < 0.01; ***, p < 0.001 (unpaired two-tailed Student’s t-test).

**Fig. S9 (for discussion)**

1. HS induces the condensate formation of CLK1, CLK3, and CLK4. HeLa cells expressing CLK1, CLK3, or CLK4 were exposed to HS (42°C, 2 hours) and subsequently subjected to heat shock recovery (37°C, 2 hours). Scale bars: 10 µm.
2. The intrinsic disorder arrangement of CLK1, CLK3, CLK4. The y-axis displays the PONDER VSL2 score (Ponder score), and the x-axis represents the amino acid position. The IDR is indicated by a blue line. Scale bars: 10 µm.
3. The IDRs derived from CLK1, CLK3, and CLK4 exhibited phase separation capability in the OptoDroplet assay. Each IDR was fused with mCherry and Cry2, and the constructs were transfected into HeLa cells. Images were captured at the indicated time after stimulation. Scale bars, 10 µm.

Panel A and C: The percentage of cells exhibiting nuclear puncta was quantified, and the data are presented as the mean ± SD from three independent experiments. ***, p < 0.001 (unpaired two-tailed Student’s t-test).

1. Amino acid sequence alignment of the kinase domains within the CLK family. The equivalent CLK2 T343 residue in the other CLK family members is denoted by a blue star.
2. The threonine residue as denoted in panel B within CLK1, CLK3, or CLK4 was mutated to alanine (TAmut). These constructs were transfected into HeLa cells. The TAmut indicates T342A, T337A, and T340A mutation for CLK1, CLK3, and CLK4, respectively.

**Supplemental tables (Table S1-10)**

**Table S1** The activated IR events unique to T343A expressing HeLa cells (CLK2_IR).

**Table S2** The details for the validated 12 activated CLK2_IR events and the RT-PCR primers for detection of these events and expression of the associated transcripts.

**Table S3** Proteins enriched in IP (control versus T343A) and IP (WT versus T343A), and the overlapping proteins between the two comparisons.

**Table S4** RNA-seq data information with SF knockdown from the ENCODE database

The table includes sample information detailing the knockdown cells with the specified SF and their respective control cells. Download links for the FASTQ data were also provided.

**Table S5** The activated IR events in the nuclear fraction, compared to the cytoplasmic fraction of T343A expressing HeLa cells.

**Table S6** The differentially expressed genes in the dGSCs in comparison to the GSCs.

**Table S7** The activated IR events in dGSCs in comparison to the GSCs.

**Table S8** The sequencing read information for RNA-seq samples

The number of raw and clean read and total bases, the rate for sequencing quality scores (Q20 and Q30) and GC percentages were indicated.

**Table S9** The Northern blot probe sequences

The sequences targeting the introns of the indicated IR events were amplified through PCR and were labeled with biotin.

**Table S10** The primers for the RIP-qPCR

Based on the binding peaks of U2AF1 or SRSF1, a pair of primers were designed: one primer located within the peak region, while the other was outside of the region. The transcripts with the validation primers were highlighted in yellow.
