# Supplementary material for: CLK2 Condensates Reorganize Nuclear Speckles and Induce Intron Retention
Source: Adv Sci (Weinh). 2024 Aug 9;11(38):2309588. doi: 10.1002/advs.202309588 (PMC11481226; doi:10.1002/advs.202309588)
Supplement: Supplementary file 2 — Supplemental Figures 1‐9 [file ADVS-11-2309588-s002.pdf]

ADVANCED SCIENCE
Open Access

Supporting Information

for *Adv. Sci.*, DOI 10.1002/advs.202309588

CLK2 Condensates Reorganize Nuclear Speckles and Induce Intron Retention

Bing Wang, Jing Li, Yanyang Song, Xuhui Qin, Xia Lu, Wei Huang, Chentai Peng, Jinxia Wei, Donghui Huang and Wei Wang**

Fig. S1

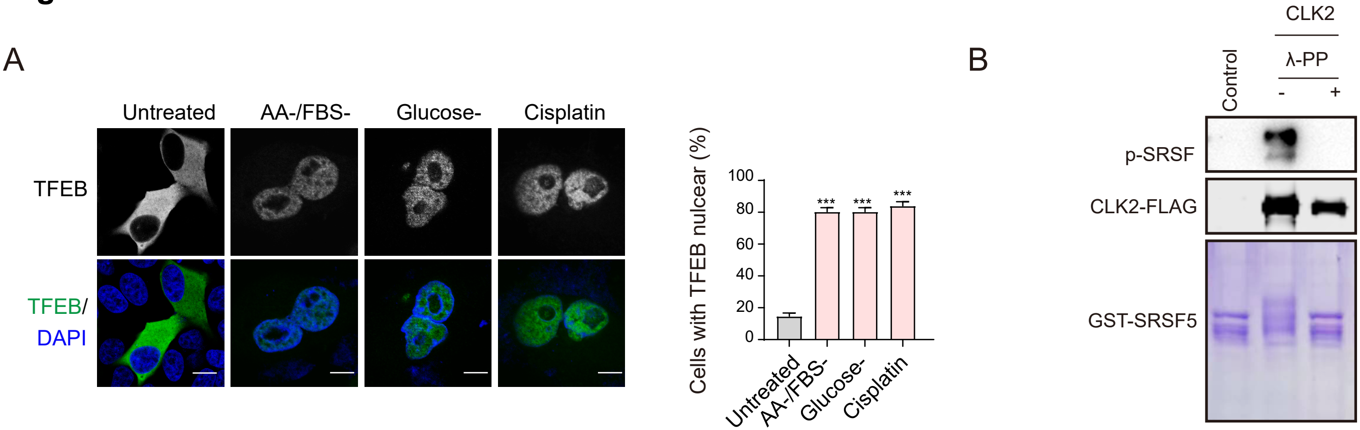
A
Untreated
AA-/FBS-
Glucose-
Cisplatin
TFEB
TFEB/
DAPI
Cells with TFEB nulcear (%)
100
80
60
40
20
0
***
***
***
Untreated
AA-/FBS-
Glucose-
Cisplatin
B
CLK2
Control
λ-PP
-
+
p-SRSF
CLK2-FLAG
GST-SRSF5

Fig. S2

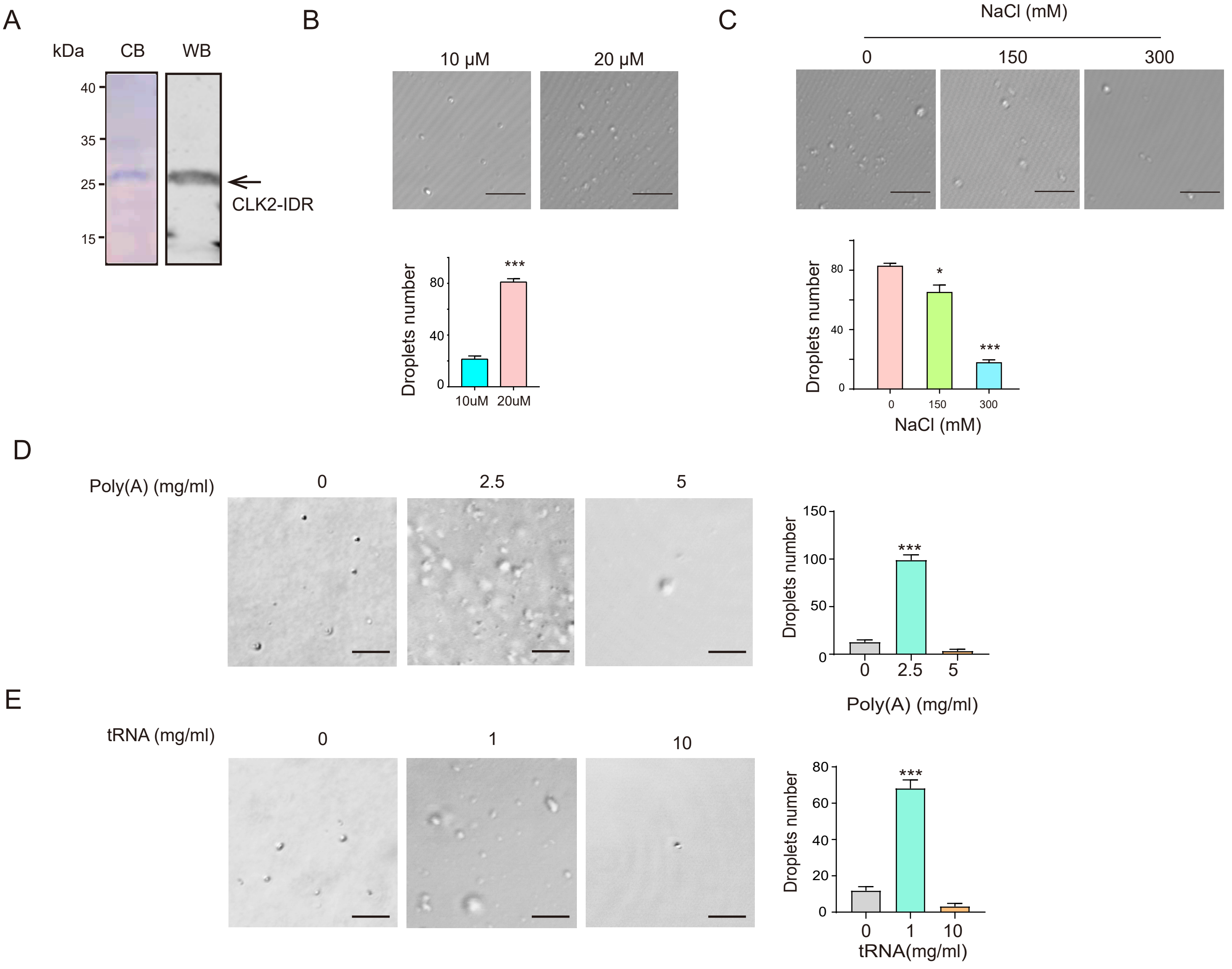

A
kDa
CB
WB
40
35
25
15
CLK2-IDR
B
10 μM
20 μM
Droplets number
80
40
0
***
10uM
20uM
C
NaCl (mM)
0
150
300
Droplets number
80
40
0
*
***
0
150
300
NaCl (mM)
D
Poly(A) (mg/ml)
0
2.5
5
Droplets number
150
100
50
0
***
0
2.5
5
Poly(A) (mg/ml)
E
tRNA (mg/ml)
0
1
10
Droplets number
80
60
40
20
0
***
0
1
10
tRNA(mg/ml)

Fig. S3

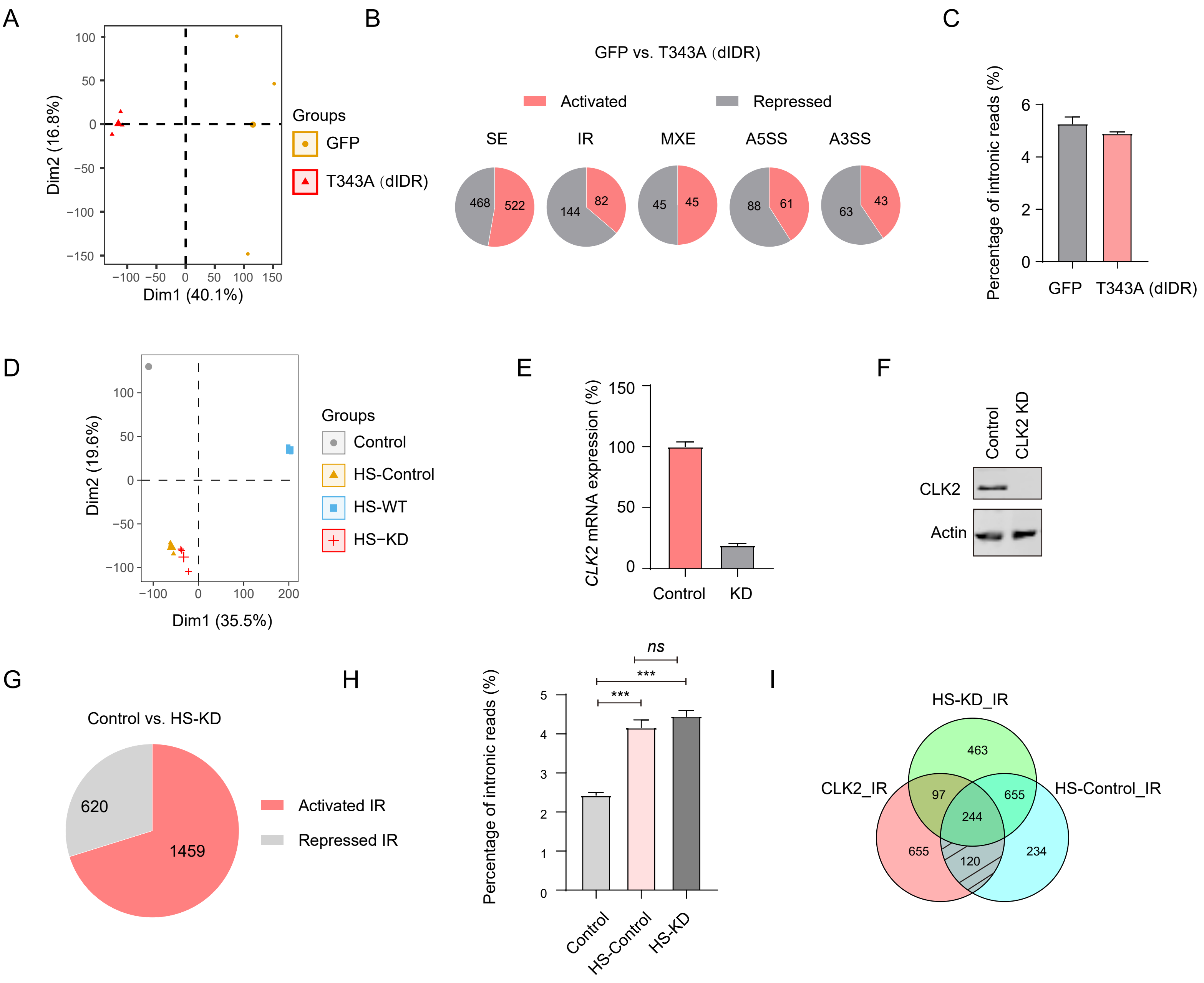
A
Dim2 (16.8%)
Dim1 (40.1%)
100
50
0
−50
−100
−150
−100 −50 0 50 100 150
Groups
GFP
T343A (dIDR)
B
GFP vs. T343A (dIDR)
Activated
Repressed
SE
IR
MXE
A5SS
A3SS
468
522
144
82
45
45
88
61
63
43
C
Percentage of intronic reads (%)
6
4
2
0
GFP
T343A (dIDR)
D
Dim2 (19.6%)
Dim1 (35.5%)
100
50
0
−50
−100
−100 0 100 200
Groups
Control
HS-Control
HS-WT
HS−KD
E
CLK2 mRNA expression (%)
150
100
50
0
Control
KD
F
Control
CLK2 KD
CLK2
Actin
G
Control vs. HS-KD
620
1459
Activated IR
Repressed IR
H
Percentage of intronic reads (%)
ns
***
***
5
4
3
2
1
0
Control
HS-Control
HS-KD
I
HS-KD_IR
CLK2_IR
HS-Control_IR
463
97
655
244
655
120
234

Fig. S4

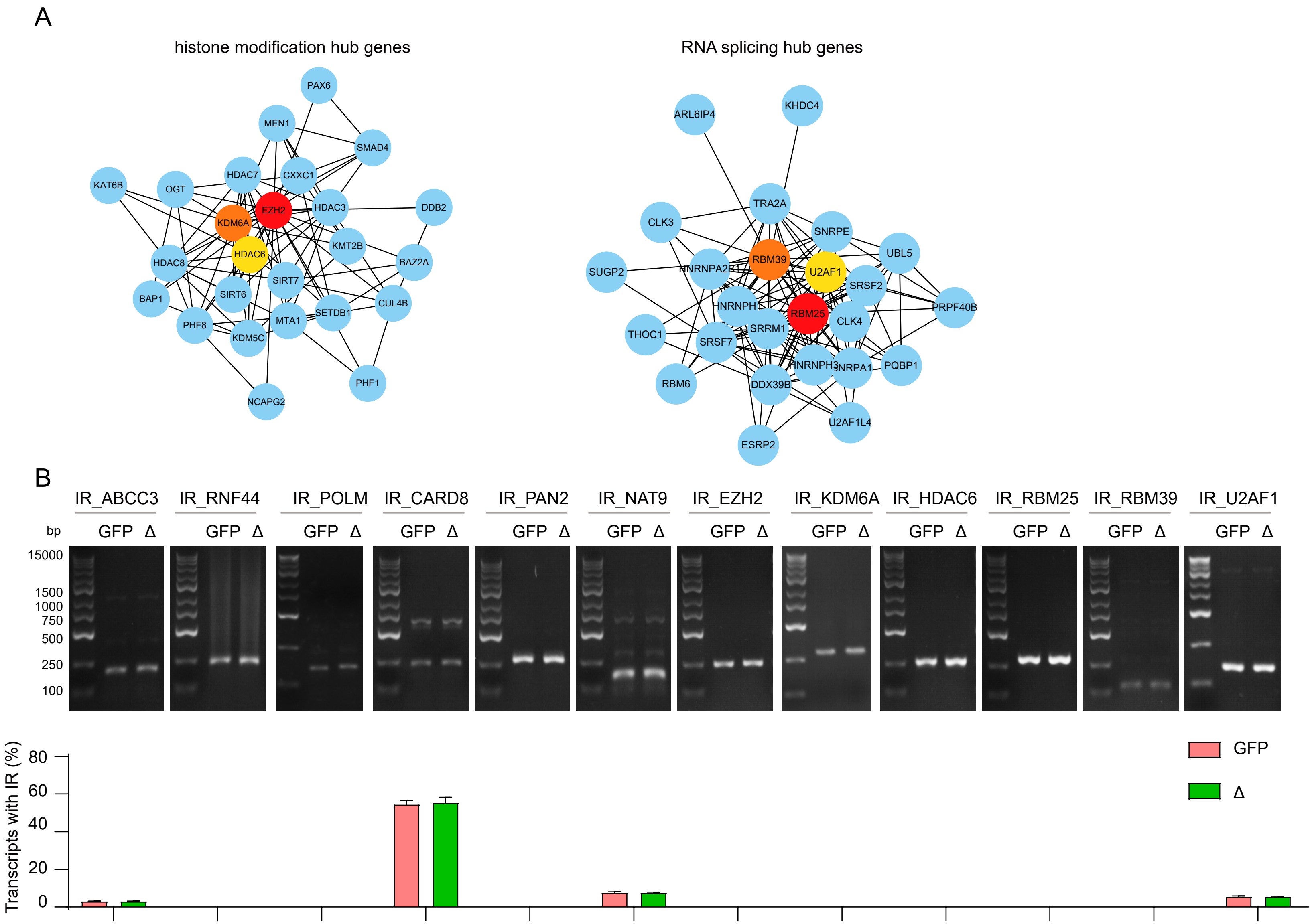
A
histone modification hub genes
PAX6
MEN1
SMAD4
KAT6B
OGT
HDAC7
CXXC1
EZH2
KDM6A
HDAC3
DDB2
KMT2B
HDAC6
HDAC8
BAZ2A
SIRT7
SIRT6
BAP1
CUL4B
SETDB1
MTA1
PHF8
KDM5C
PHF1
NCAPG2
RNA splicing hub genes
ARL6IP4
KHDC4
TRA2A
CLK3
SNRPE
UBL5
RBM39
U2AF1
SUGP2
HNRNPA2B1
SRSF2
HNRNPH1
RBM25
PRPF40B
CLK4
THOC1
SRRM1
SRSF7
HNRNPH3
HNRNPA1
PQBP1
RBM6
DDX39B
U2AF1L4
ESRP2
B
IR_ABCC3
IR_RNF44
IR_POLM
IR_CARD8
IR_PAN2
IR_NAT9
IR_EZH2
IR_KDM6A
IR_HDAC6
IR_RBM25
IR_RBM39
IR_U2AF1
bp
GFP Δ
15000
1500
1000
750
500
250
100
Transcripts with IR (%)
80
60
40
20
0
GFP
Δ

Fig. S5

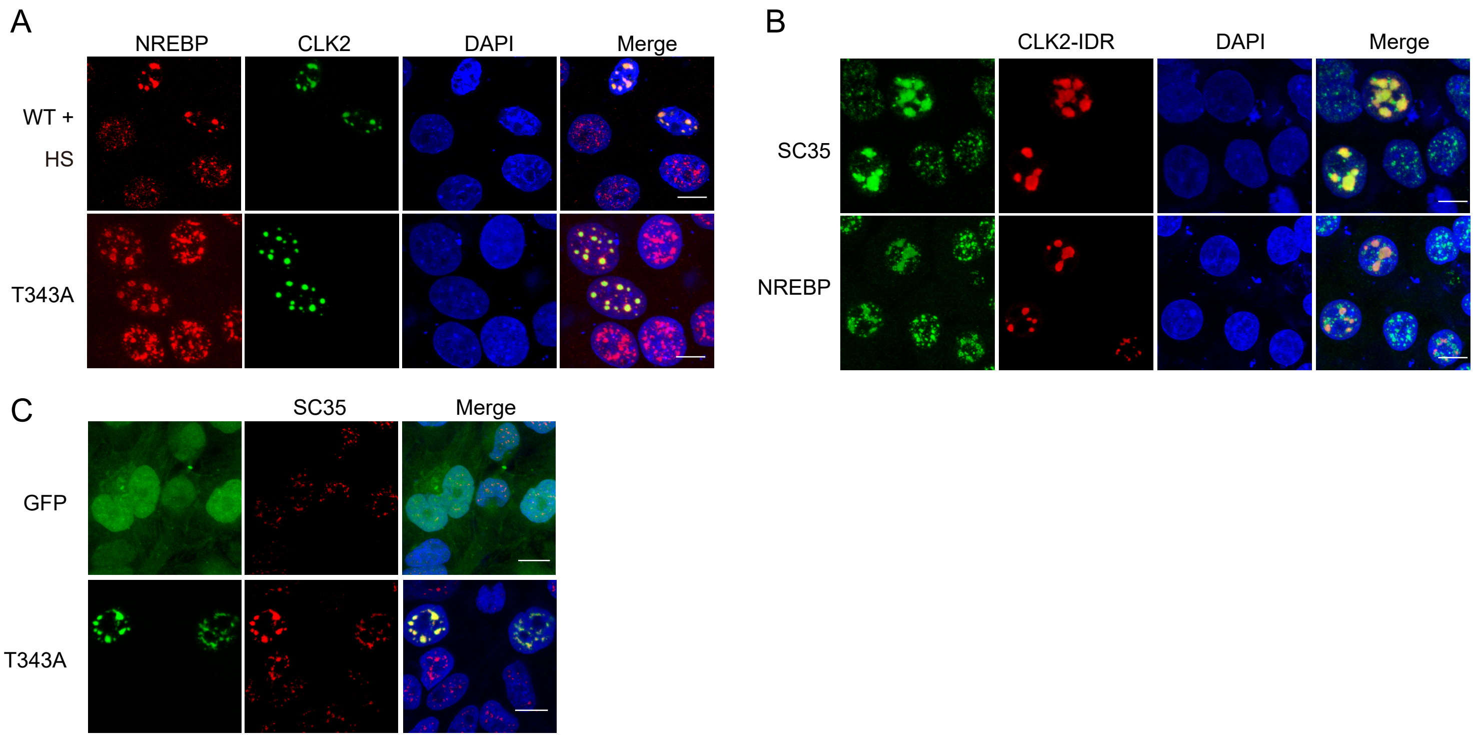
A
NREBP
CLK2
DAPI
Merge
WT +
HS
T343A
B
CLK2-IDR
DAPI
Merge
SC35
NREBP
C
SC35
Merge
GFP
T343A

Fig. S6

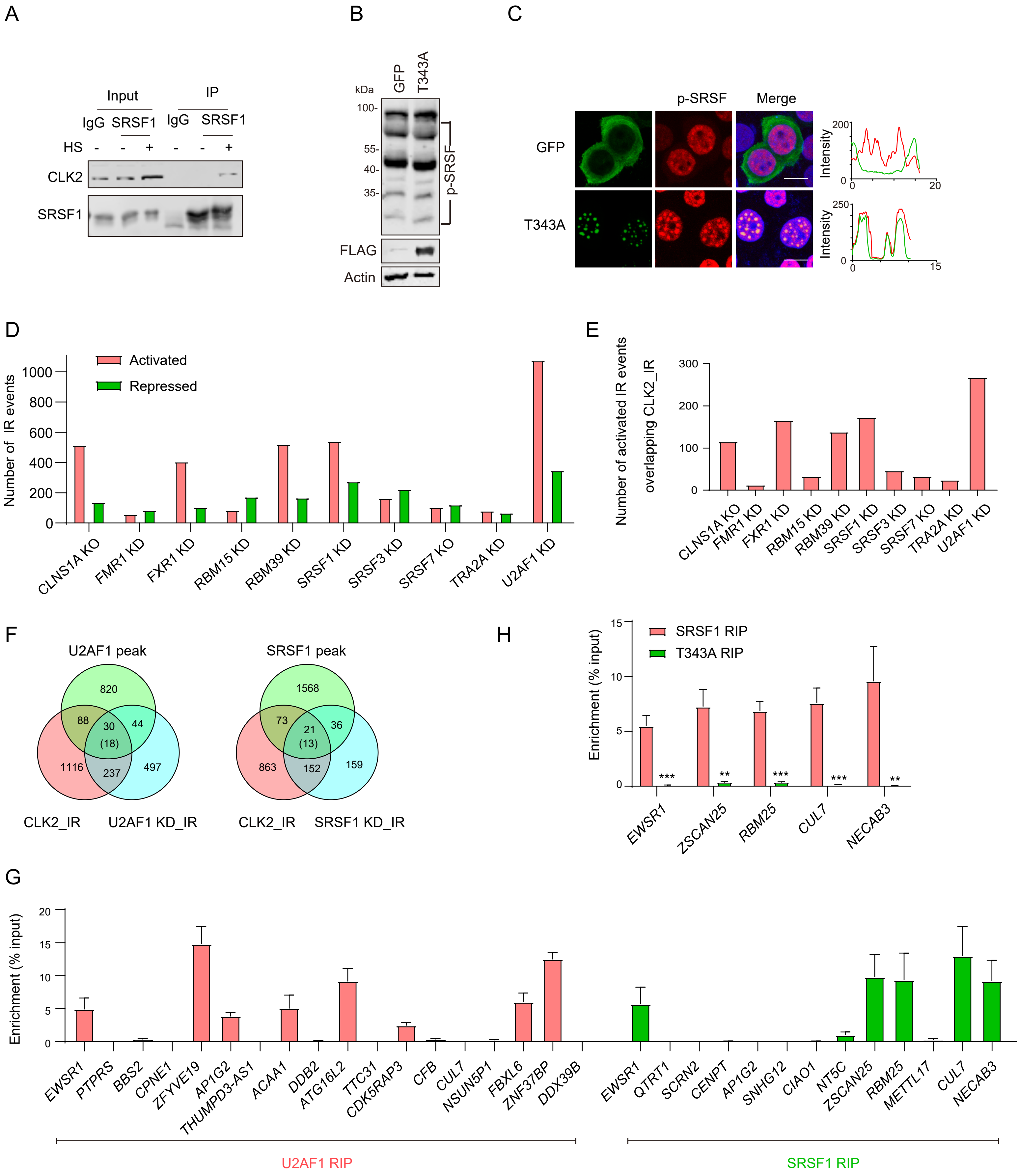
A
Input
IP
IgG
SRSF1
HS
CLK2
B
kDa
GFP
T343A
100-
55-
40-
35-
p-SRSF
FLAG
Actin
C
p-SRSF
Merge
GFP
T343A
Intensity
200
250
0
20
15
D
Activated
Repressed
Number of IR events
1000
800
600
400
200
0
CLNS1A KO
FMR1 KD
FXR1 KD
RBM15 KD
RBM39 KD
SRSF1 KD
SRSF3 KD
SRSF7 KO
TRA2A KD
U2AF1 KD
E
Number of activated IR events
overlapping CLK2_IR
300
200
100
0
F
U2AF1 peak
820
88
30
(18)
44
1116
237
497
CLK2_IR
U2AF1 KD_IR
SRSF1 peak
1568
73
21
(13)
36
863
152
159
CLK2_IR
SRSF1 KD_IR
H
SRSF1 RIP
T343A RIP
Enrichment (% input)
15
10
5
0
***
**
***
***
**
EWSR1
ZSCAN25
RBM25
CUL7
NECAB3
G
Enrichment (% input)
20
15
10
5
0
EWSR1
PTPRS
BBS2
CPNE1
ZFYVE19
AP1G2
THUMPD3-AS1
ACAA1
DDB2
ATG16L2
TTC31
CDK5RAP3
CFB
CUL7
NSUN5P1
FBXL6
ZNF37BP
DDX39B
EWSR1
QTRT1
SCRN2
CENPT
AP1G2
SNHG12
CIAO1
NT5C
ZSCAN25
RBM25
METTL17
CUL7
NECAB3
U2AF1 RIP
SRSF1 RIP

Fig. S7

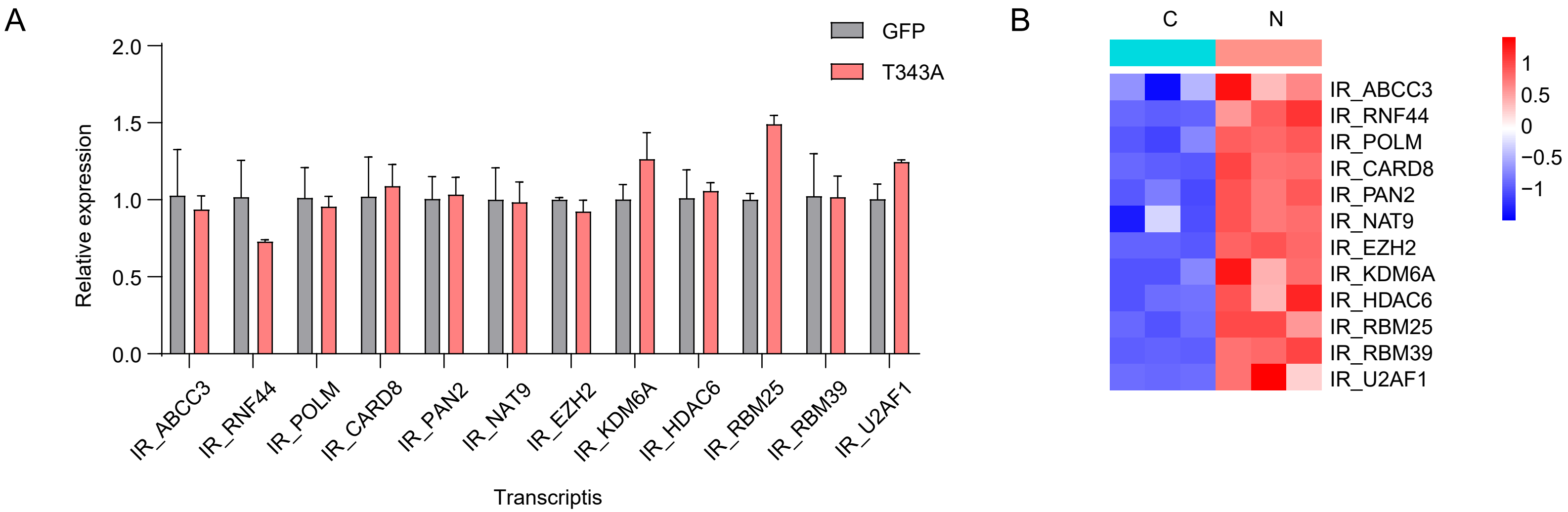
A
GFP
T343A
2.0
1.5
1.0
0.5
0.0
Relative expression
IR_ABCC3
IR_RNF44
IR_POLM
IR_CARD8
IR_PAN2
IR_NAT9
IR_EZH2
IR_KDM6A
IR_HDAC6
IR_RBM25
IR_RBM39
IR_U2AF1
Transcriptis
B
C
N
IR_ABCC3
IR_RNF44
IR_POLM
IR_CARD8
IR_PAN2
IR_NAT9
IR_EZH2
IR_KDM6A
IR_HDAC6
IR_RBM25
IR_RBM39
IR_U2AF1
1
0.5
0
−0.5
−1

Fig. S8

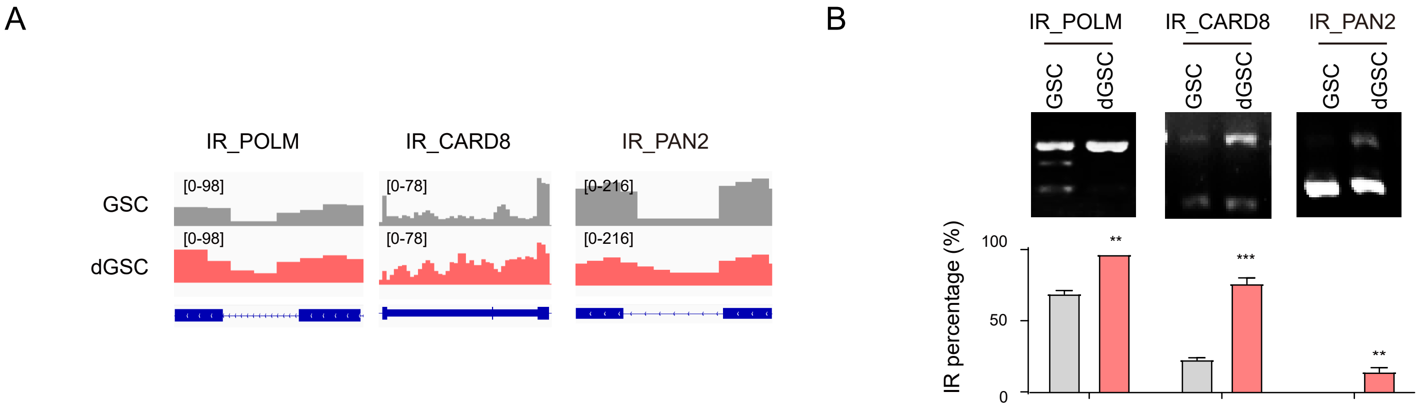

A
IR_POLM
IR_CARD8
IR_PAN2
GSC
dGSC
[0-98]
[0-98]
[0-78]
[0-78]
[0-216]
[0-216]
B
IR_POLM
IR_CARD8
IR_PAN2
GSC
dGSC
GSC
dGSC
GSC
dGSC
IR percentage (%)
100
50
0
**
***
**

Fig. S9

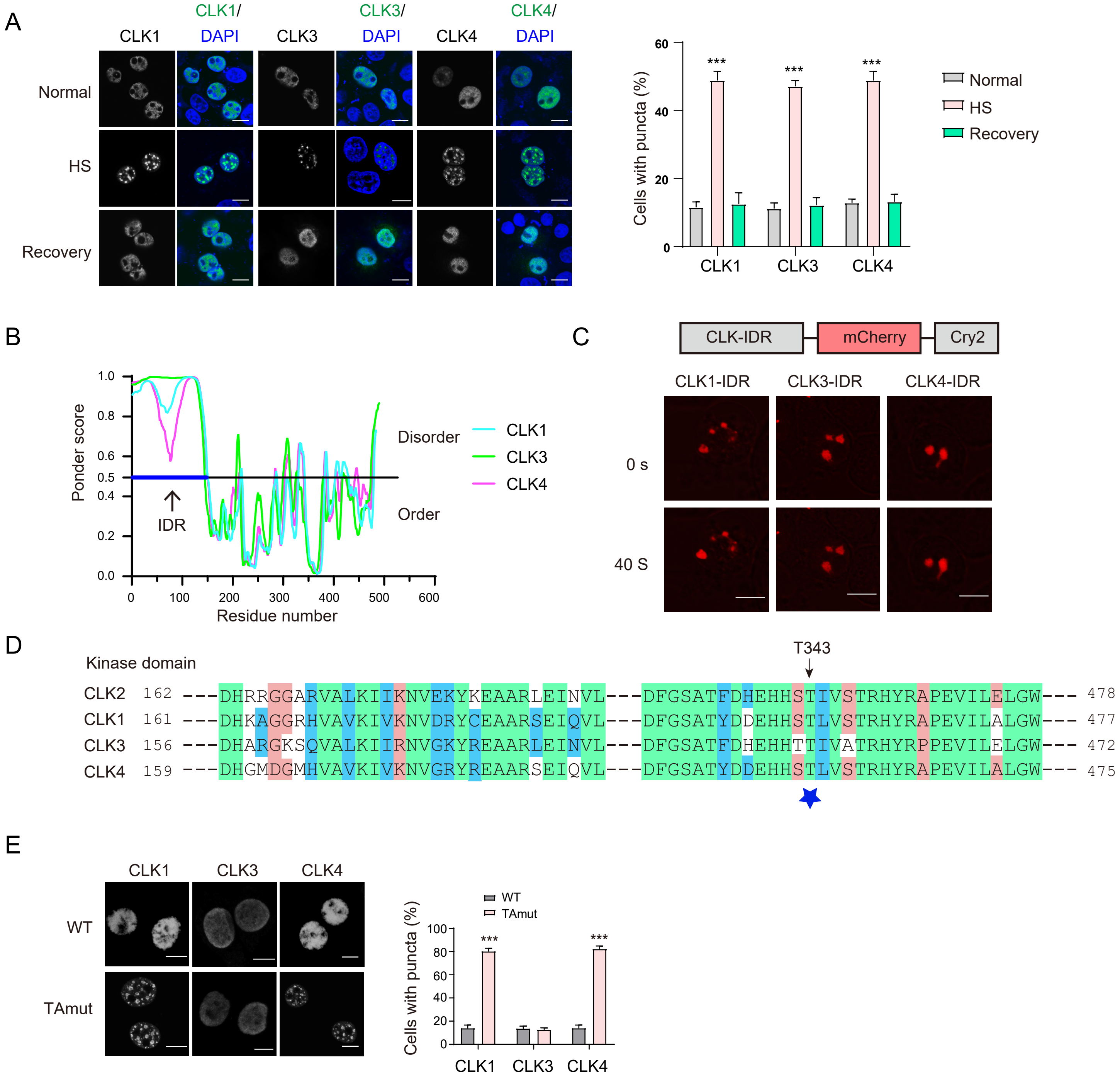

A
CLK1
CLK1/
DAPI
CLK3
CLK3/
DAPI
CLK4
CLK4/
DAPI
Normal
HS
Recovery
Cells with puncta (%)
60
40
20
0
***
***
***
CLK1
CLK3
CLK4
Normal
HS
Recovery
B
Ponder score
1.0
0.8
0.6
0.5
0.4
0.2
0.0
0
100
200
300
400
500
600
Residue number
IDR
Disorder
Order
CLK1
CLK3
CLK4
C
CLK-IDR
mCherry
Cry2
CLK1-IDR
CLK3-IDR
CLK4-IDR
0 s
40 S
D
Kinase domain
T343
CLK2 162 ---DHRRGGARVALKIIKNVEKYKEAARLEINVL---DFGSATFDHEHHSTIVSTRHYRAPEVILELGW--- 478
CLK1 161 ---DHKAGGRHVAVKIVKNVDRYCEAARSEIQVL---DFGSATYDDEHHSTLVSTRHYRAPEVILALGW--- 477
CLK3 156 ---DHARGKSQVALKIIRNVGKYREAARLEINVL---DFGSATFDHEHHTTIVATRHYRPPEVILELGW--- 472
CLK4 159 ---DHGMDGMHVAVKIVKNVGRYREAARSEIQVL---DFGSATYDDEHHSTLVSTRHYRAPEVILALGW--- 475
E
CLK1
CLK3
CLK4
WT
TAmut
WT
TAmut
Cells with puncta (%)
100
80
60
40
20
0
***
***
CLK1
CLK3
CLK4
