# Supplementary material for: CLK2 Condensates Reorganize Nuclear Speckles and Induce Intron Retention
Source: Adv Sci (Weinh). 2024 Aug 9;11(38):2309588. doi: 10.1002/advs.202309588 (PMC11481226; doi:10.1002/advs.202309588)

FIg. 1 western blot raw data

1E

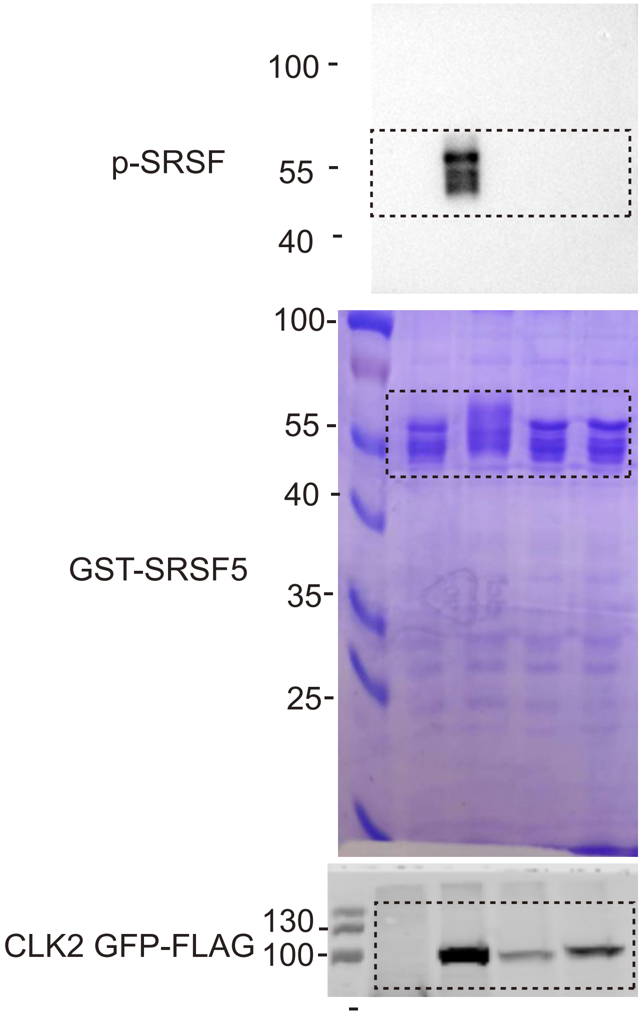

1F

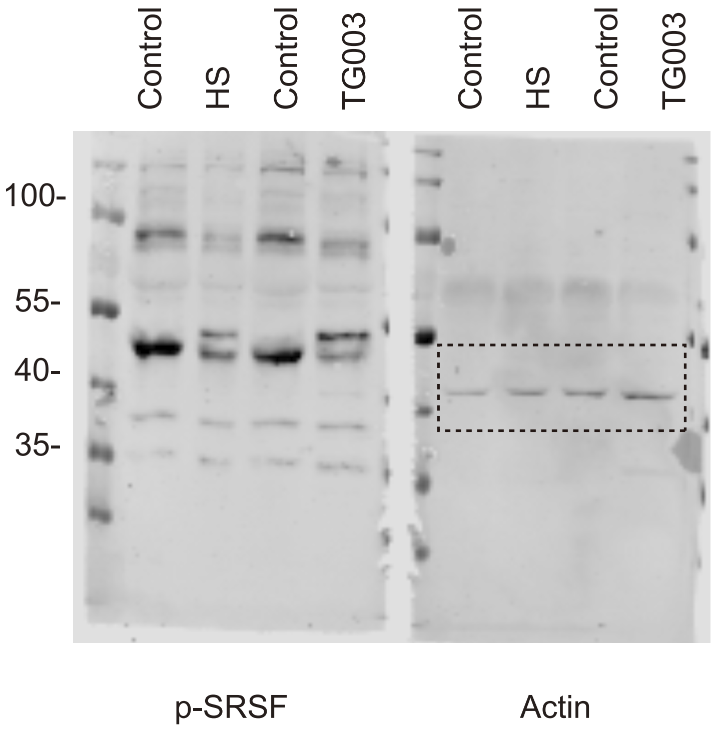

1H

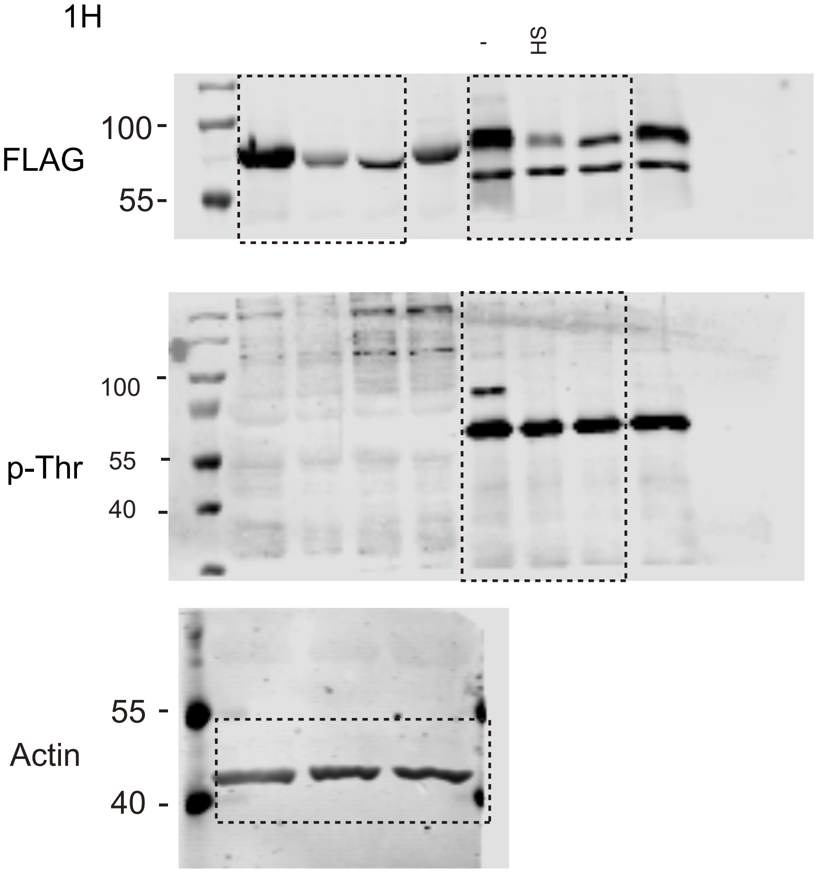

Fig. 2 western blot raw data

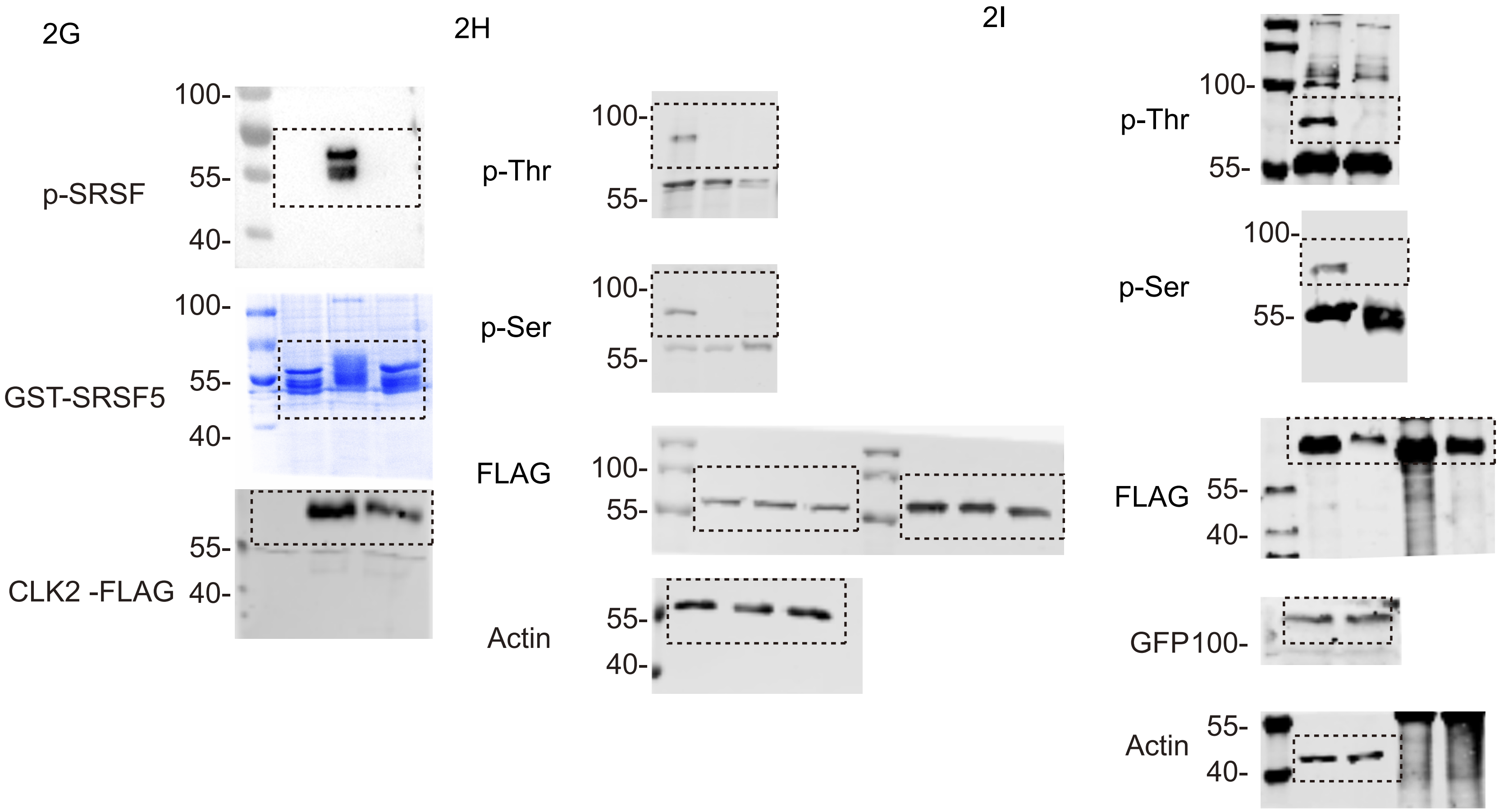

Fig. 4 Northern blot raw data

4H

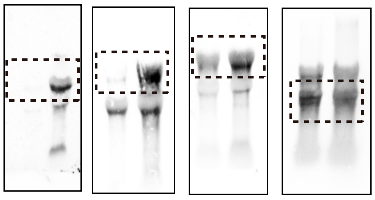

FIg. 5 western blot raw data

5D

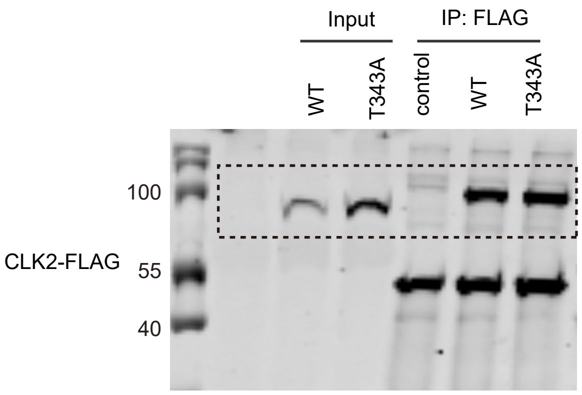

FIg. 6 western blot raw data

GFP
100
55
40

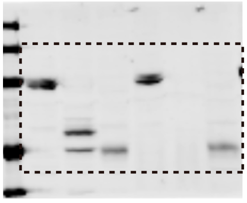

GFP
100
55
40

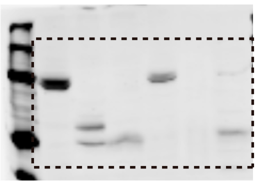

FLAG
100
55
40

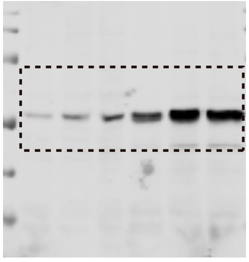

FLAG
100
55
40

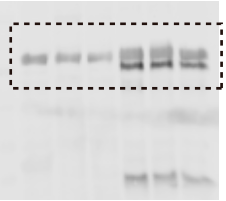

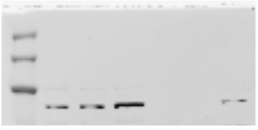

GFP GFP CLK2 T343A GFP GFP CLK2 T343A

40-
35-

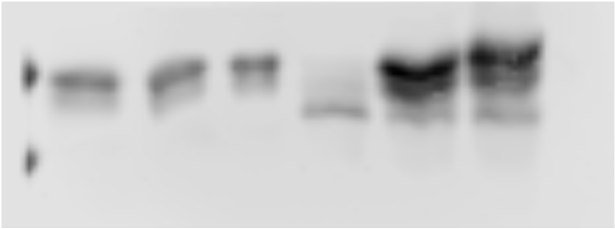

Input Anti-SRSF1 IgG IP Anti-SRSF1

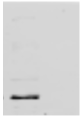

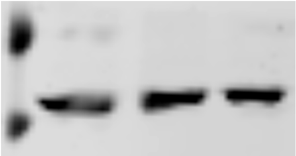

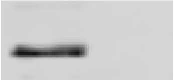

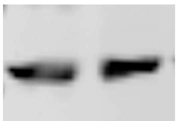

FIg. 8 western blot raw data

FIg. 8D

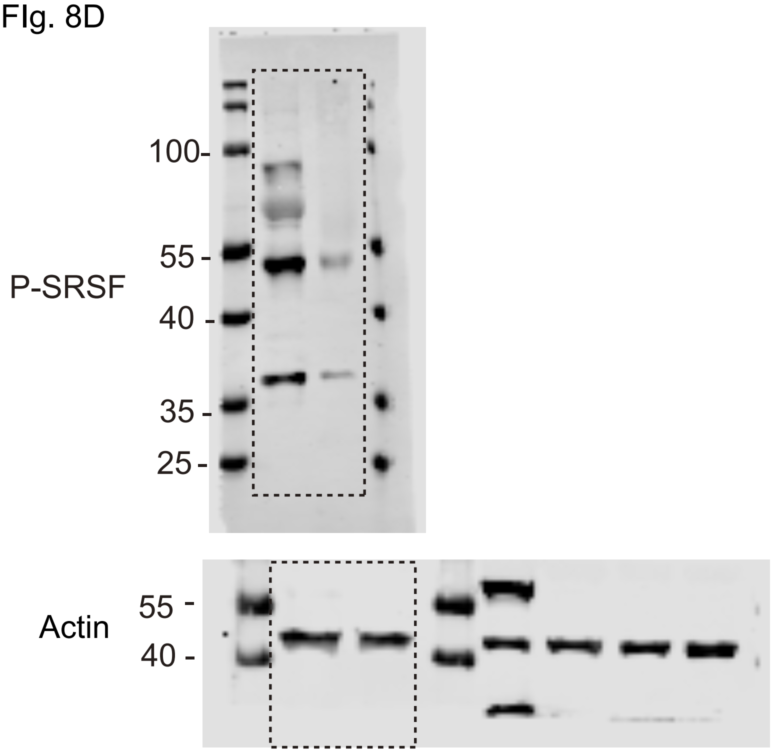

FIg. 8E

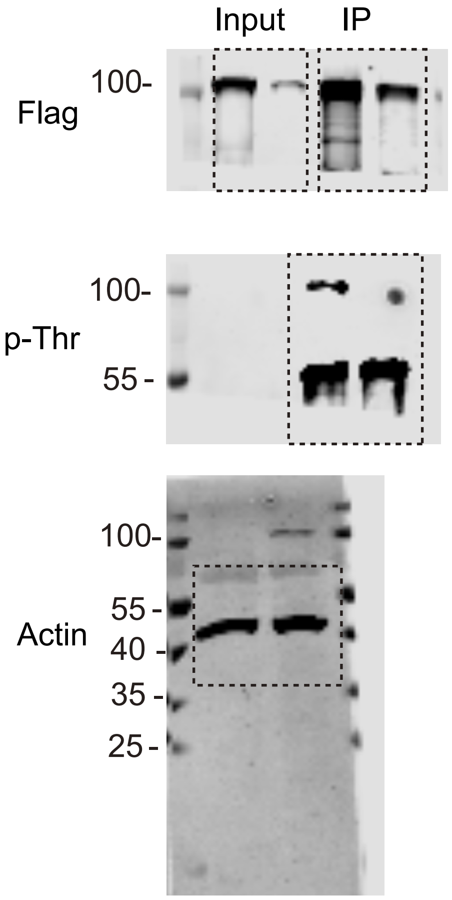

Fig. S2A

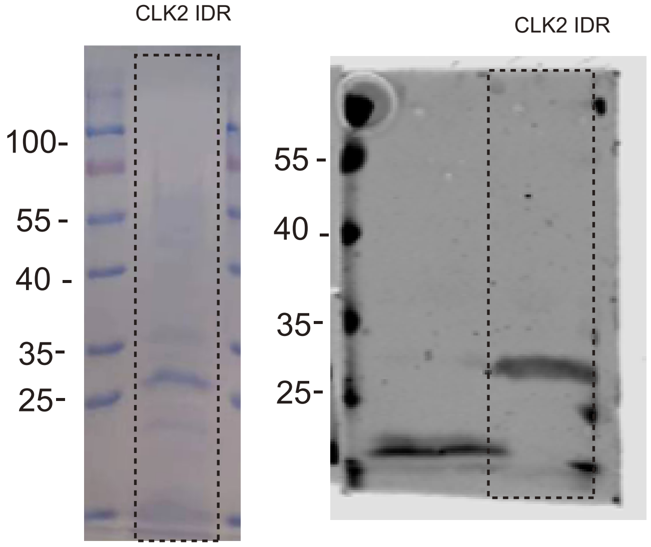

Fig. S3G

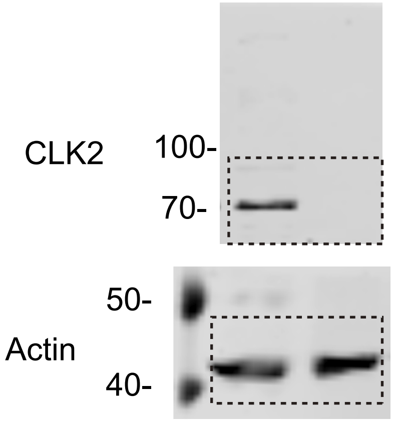

Fig. S6A

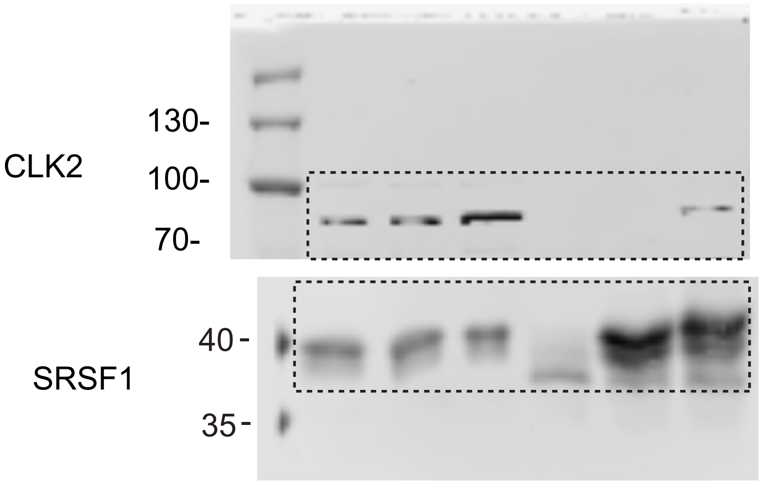

Fig. S6B

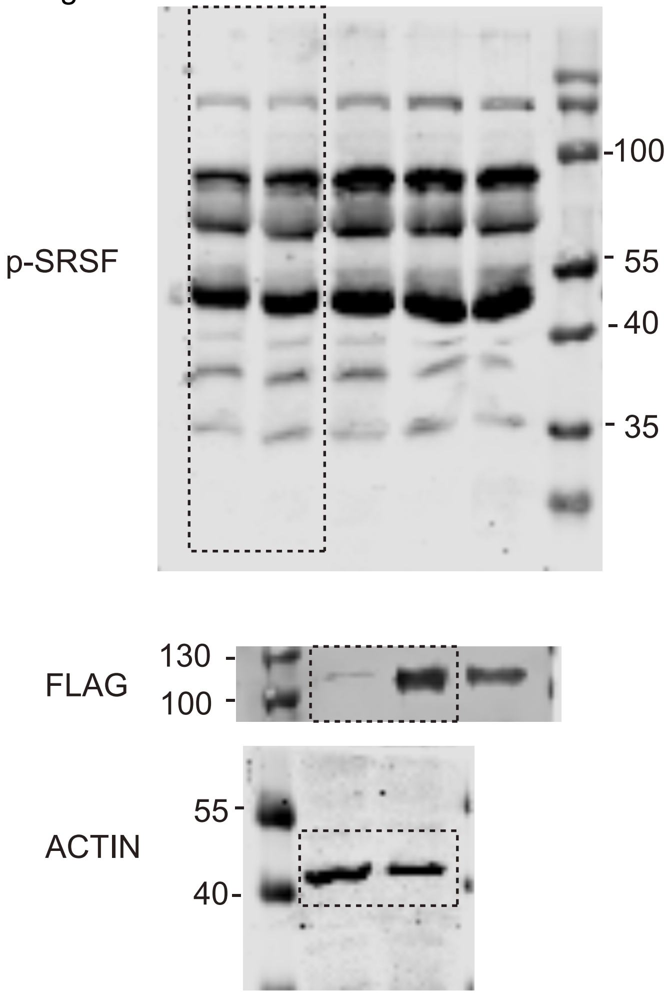

Supplement: Supplementary file 4 — Supplemental Figure [file ADVS-11-2309588-s001.zip › raw western blots.pdf]
